# Identification and characterization of evolutionarily conserved alternative splicing events in a mangrove genus *Sonneratia*

**DOI:** 10.1038/s41598-018-22406-6

**Published:** 2018-03-13

**Authors:** Yuchen Yang, Wuxia Guo, Xu Shen, Jianfang Li, Shuhuan Yang, Sufang Chen, Ziwen He, Renchao Zhou, Suhua Shi

**Affiliations:** 1 State Key Laboratory of Biocontrol and Guangdong Provincial Key Laboratory of Plant Resources, Sun Yat-sen University, Guangzhou, 510275 China; 2 Department of Genetics, Department of Biostatistics, Department of Computer Science University of North Carolina, Chapel Hill, NC 27599 USA

## Abstract

Alternative splicing (AS), which produces multiple mRNA transcripts from a single gene, plays crucial roles in plant growth, development and environmental stress responses. Functional significances of conserved AS events among congeneric species have not been well characterized. In this study, we performed transcriptome sequencing to characterize AS events in four common species of *Sonneratia*, a mangrove genus excellently adaptive to intertidal zones. 7,248 to 12,623 AS events were identified in approximately 25% to 35% expressed genes in the roots of the four species. The frequency of AS events in *Sonneratia* was associated with genomic features, including gene expression level and intron/exon number and length. Among the four species, 1,355 evolutionarily conserved AS (ECAS) events were identified from 1,170 genes. Compared with non-ECAS events, ECAS events are of shorter length and less possibility to introduce premature stop codons (PTCs) and frameshifts. Functional annotations of the genes containing ECAS events showed that four of the 26 enriched Gene Ontology (GO) terms are involved in proton transport, signal transduction and carbon metabolism, and 60 genes from another three GO terms are implicated in responses to osmotic, oxidative and heat stresses, which may contribute to the adaptation of *Sonneratia* species to harsh intertidal environments.

## Introduction

Alternative splicing (AS) is a general post-transcriptional regulation mechanism in eukaryotes; it can generate multiple mRNA isoforms from a single gene. AS can increase mRNA and protein diversity without a corresponding increase in gene number, which is crucial for developmental processes and responses to environmental stresses in eukaryotic organisms^1–4^. Approximately 92–94% of genes with multiple exons in human have been identified to be alternatively spliced^5^. In plants, despite being lower, the proportion of AS in intron-containing genes is still considerable. Based on transcriptome sequencing, approximately 61% of intron-containing genes have shown evidence of AS in *Arabidopsis*^2^. The proportion of genes containing AS ranges from 36% to 52.7% in *Populus trichocarpa*^6^, maize^7^, rice^8^, and soybean^9^.

Not all AS events are of functional significance. Some AS events can give rise to premature stop codons (PTCs), which are a target of non-sense-mediated decay (NMD) pathway^10–12^. NMD is an important posttranscriptional regulation mechanism that removes non-functional transcripts and controls gene expression levels. In Arabidopsis, approximately 43% alternatively spliced isoforms contain a putative NMD target. Thus, distinguishing AS events with biological roles from those without any functional significance is critical for characterizing the functions of AS in plants^1^.

Evolutionarily conserved AS (ECAS) events across different species are thought to be more selectively favored, which provides a good opportunity to infer the function of alternatively spliced isoforms^1^. Wang and Brendel first implemented a genome-wide comparative analysis of AS between *Arabidopsis* and rice, revealing that approximately 24.5% of the genes with AS events in Arabidopsis were conserved compared with rice^4^. Increasing numbers of ECAS events are being identified and/or functionally characterized^3,6,13–16^. However, previous studies have mainly focused on ECAS events between highly divergent species from different classes (*Arabidopsis* and rice)^4,15^, different tribes (*Medicago truncatula* and *Lotus japonicas*)^16^ or genera from the same family (*Arabidopsis* and *Brassica*)^14^, or ECAS events between slightly diverged lines of the same species (*P. trichocarpa*)^6^. The extent and features of ECAS events between recently diverged congeneric species, however, have not been comprehensively investigated in plants^17^.

In this study, we sought to address this issue by using four species of *Sonneratia* (Lythraceae *sensu lato*) as a study system. *Sonneratia*, a genus of mangroves, is one of the most important components of intertidal zones of the tropical and subtropical coasts in the Indo West Pacific region^18^. This genus consists of six species^19,20^, among which four species, namely, *S. alba*, *S. caseolaris*, *S. ovata* and *S. apetala* are relatively common^21^. Among the four species, *S. caseolaris* is the most divergent species, *S. ovata* is sister to *S. apetala*, and *S. alba* is sister to the clade including *S. ovata* and *S. apetala*^22,23^. All species of this genus are well adapted to harsh intertidal zones characterized by high salinity, hypoxia, ultraviolet (UV) radiation, although they have slight differences in their positions in intertidal zones^21^. Recent studies based on transcriptome sequencing suggested that transcriptional regulation plays crucial roles for *S. alba*, as well as other mangrove species, surviving in extreme intertidal habitats^24–31^. However, being non-model plants, mangrove plants have lacked the genomic resources necessary for comprehensively characterizing AS events in the past. The genome of *S. alba* has been sequenced recently (He *et al*., unpublished), owing to the advent of high-throughput sequencing technologies.

In this study, we identified and compared AS events in the four common species of *Sonneratia* based on transcriptomic data obtained by Illumina sequencing. We wish to address the following questions: (1) Whether the gene expression level and intron/exon number and length can affect the frequencies of AS events in *Sonneratia* species? (2) What are the features of the ECAS events compared with non-evolutionarily conserved AS (non-ECAS) events? and (3) What are the putative functions of the genes containing ECAS events? We hope that our study can enrich our understanding of the conservation of AS in different species within a genus and shed light on the genetic connection between functional AS events and evolutionary adaptation in *Sonneratia*.

## Results

### Detection of alternative splicing (AS) events in four species of *Sonneratia*

To explore and compare alternative splicing patterns in four *Sonneratia* species (*S. alba*, *S. ovata*, *S. apetala* and *S. caseolaris*) at the genomic level, we performed high-throughput RNA-seq for root tissues. Raw reads of the four transcriptomes have been submitted to the Sequence Read Archive of NCBI with accession numbers SRP068401 (*S. alba*: SRS1246730; *S. caseolaris*: SRS1246739; *S. apetala*: SRS1246741; *S. ovata*: SRS1246742). In total, approximately 15.51 to 49.37 million paired-end reads of 75 or 90 bp in length were obtained from the four species (Table 1). After data trimming and filtering, 11.72, 23.22, 24.62 and 38.57 million high-quality reads were mapped to the *S. alba* genome (EMBL accession number PRJEB8424) for the four species. In each species, over 60% of reads were uniquely aligned to the reference genome, and as expected, the majority of these reads were mapped to annotated exonic regions. 22,954 to 24,167 genes were covered by the aligned reads of the four species, accounting for over 80% of the total number of genes of *S. alba*. A prerequisite for a comprehensive survey of alternative splicing (AS) is the ability to reliably detect splice junctions (SJs). To investigate potential AS events, we first identified all SJs with conservative criteria (see “Methods” for detail). 77,351, 94,432, 94,596 and 103,127 SJs were identified from 14,298, 15,784, 15,213 and 15,787 intron-containing genes in *S. alba*, *S. ovata*, *S. apetala* and *S. caseoalris*, respectively. We found that, in all four species, the majority of SJs (>98%) resided in annotated genes and were mainly located in coding sequence (CDS) regions of protein coding genes (Supplementary Fig. S1).

**Table 1:** Alignment statistics for the transcriptome data of four *Sonneratia* species.

Species	Read length (bp)	No. of raw reads (million)	No. of filtered reads (million)	No. (proportion) of aligned reads (million)	No. (proportion) of uniquely aligned reads (million)	No. of mapped genes
*S. alba*	75	15.51	11.72	10.50 (89.62%)	7.30 (62.25%)	22954
*S. ovata*	90	26.18	23.22	19.98 (86.05%)	16.49 (71.02%)	24167
*S. apetala*	90	27.70	24.63	19.71 (80.05%)	16.13 (65.51%)	23828
*S. caseolaris*	75	49.37	38.57	31.23 (80.95%)	24.60 (63.77%)	23976

No., number.

**Figure 1:**
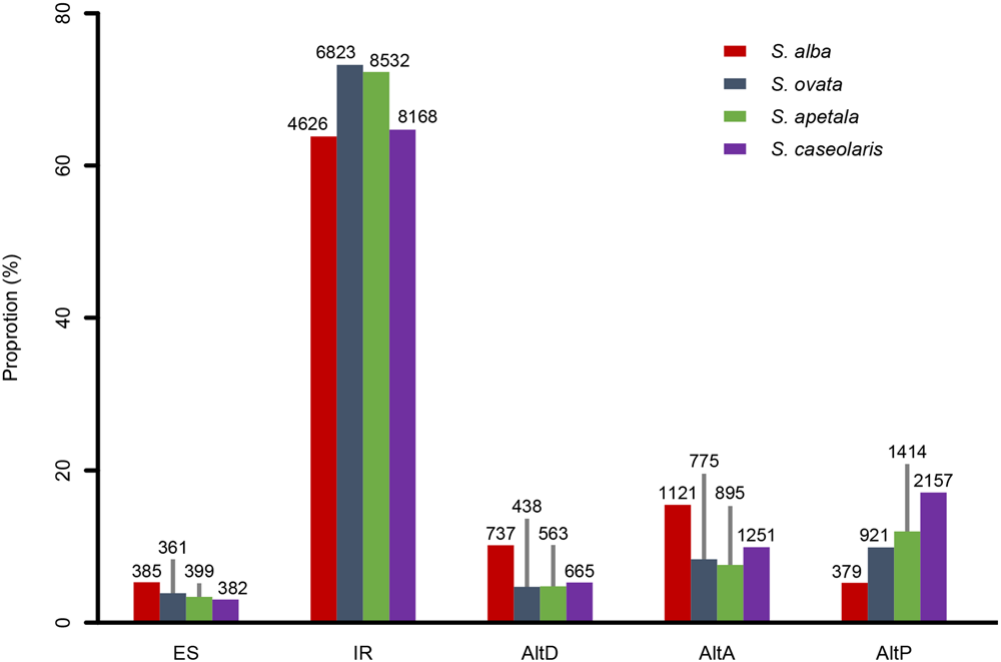
Distributions of alternative splicing (AS) types in four *Sonneratia* species.

**Figure 2:**
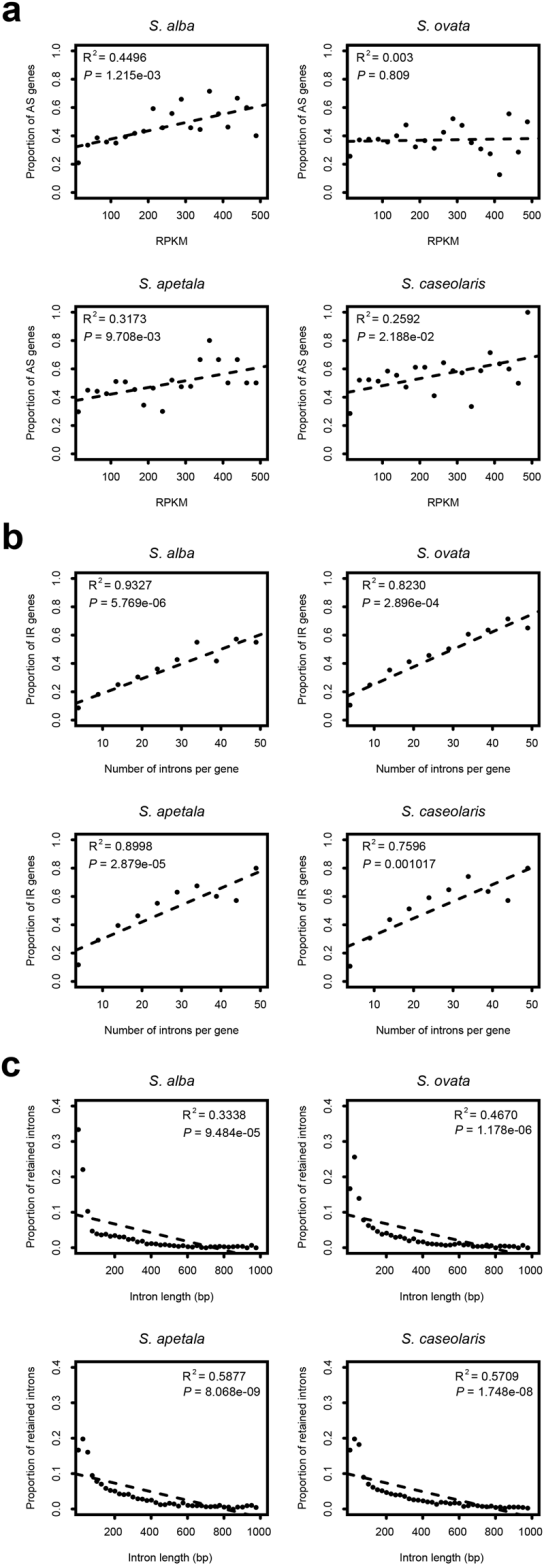
Correlations between the proportion of alternative splicing (AS) events and genic features in the four *Sonneratia* species. (**a)** Correlations between the proportion of AS events and the gene expression level of genes in the four *Sonneratia* species. **(b)** Correlations between the proportion of intron retention (IR) events and the intron number per gene in the four *Sonneratia* species. **(c)** Correlations between the proportion of IR events and the intron length of genes in the four *Sonneratia* species.

In total, we detected 7,248, 9,318, 11,803, and 12,623 AS events, in 25.16%, 28.40%, 33.24% and 34.62% of expressed genes from *S. alba*, *S. ovata*, *S. apetala* and *S. caseolaris*, respectively (Supplementary Table S1). Intron retention (IR) was the most common AS event in all the four *Sonneratia* species with proportions ranging from 63.82% to 73.22% (Fig. 1; Supplementary Table S1). However, the three other AS types varied in proportion among the four species. For example, the second most common AS type in *S. alba* was alternative acceptor (AltA, 15.47%), while it was alternative position (AltP) in the three other species (9.88–17.09%). We also tested for the correlations between AS occurrence and genic features in each species of *Sonneratia*. The occurrence of AS was positively correlated with the gene expression level in three of the four species (t-test; *P* < 0.05 for *S. alba*, *S. apetala* and *S. caseolaris*, and *P* = 0.809 for *S. ovata*, Fig. 2a). The occurrence of IR events showed a significantly positive correlation with intron number (*P* < 0.01, Fig. 2b), but a significantly negative correlation with intron length in all four species (*P* < 0.001, Fig. 2c). Positive and negative correlations were also observed between the occurrence of exon skipping (ES) events and the number of exons (*P* < 0.05, Supplementary Fig. S2a), and between the occurrence of ES events and exon length (*P* < 0.001, Supplementary Fig. S2b), respectively.

### Identification of evolutionarily conserved alternative splicing (ECAS) events in *Sonneratia*

**Figure 3:**
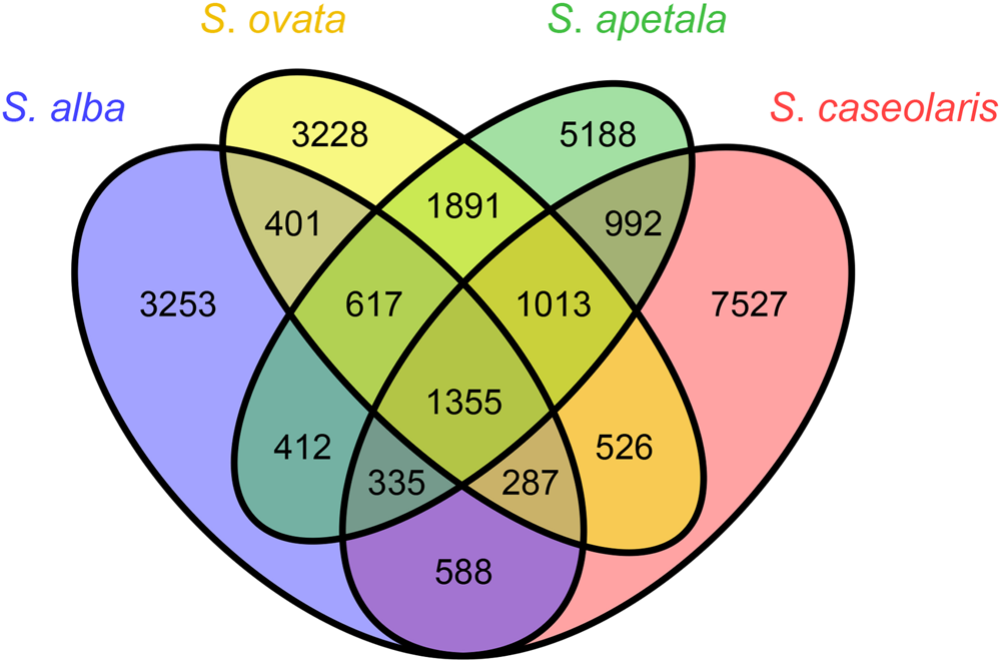
Shared and private alternative splicing (AS) events in four *Sonneratia* species.

The overall statistics of shared/unique AS events for the four *Sonneratia* species are shown in Fig. 3. We found that evolutionarily conserved AS (ECAS) events varied widely in different species pairs/groups. In the analysis of ECAS events between species pair of *Sonneratia*, there was no significant correlation between the proportion of ECAS and sequence divergence (*P* = 0.092, Supplementary Fig. S3). The largest number of ECAS events was observed between *S. apetala* and *S. ovata* (4,876), while the smallest number was observed between *S. alba* and *S. caseolaris* (2,565). Correspondingly, the highest proportion of ECAS events was identified between *S. ovata* and *S. apetala* (30.01%), while 19.13% and 16.65% of events existed between *S. alba* and *S. ovata* and between *S. alba* and *S. apetala*, respectively (Supplementary Table S2). In contrast, we detected relatively low levels of conserved AS events between *S. caseolaris* and the other three species, which ranged from 14.82 to 17.82%. With regard to the three-species analysis, the highest proportion of ECAS events (9.82%) were detected in *S. alba* - *S. ovata* - *S. apetala*, followed by *S. ovata* - *S. apetala* - *S. caseolaris* (9.7%). The last two groups, *S. alba* - *S. apetala* - *S. caseolaris* (6.93%) and *S. alba* - *S. ovata* - *S. caseolaris* (7.32%), had similar proportions of ECAS events (Supplementary Table S2). Among all four *Sonneratia* species, 1,355 ECAS events were identified from 1,170 genes (Supplementary Table S3).

### Features of evolutionarily conserved alternative splicing (ECAS) events in *Sonneratia*

**Figure 4:**
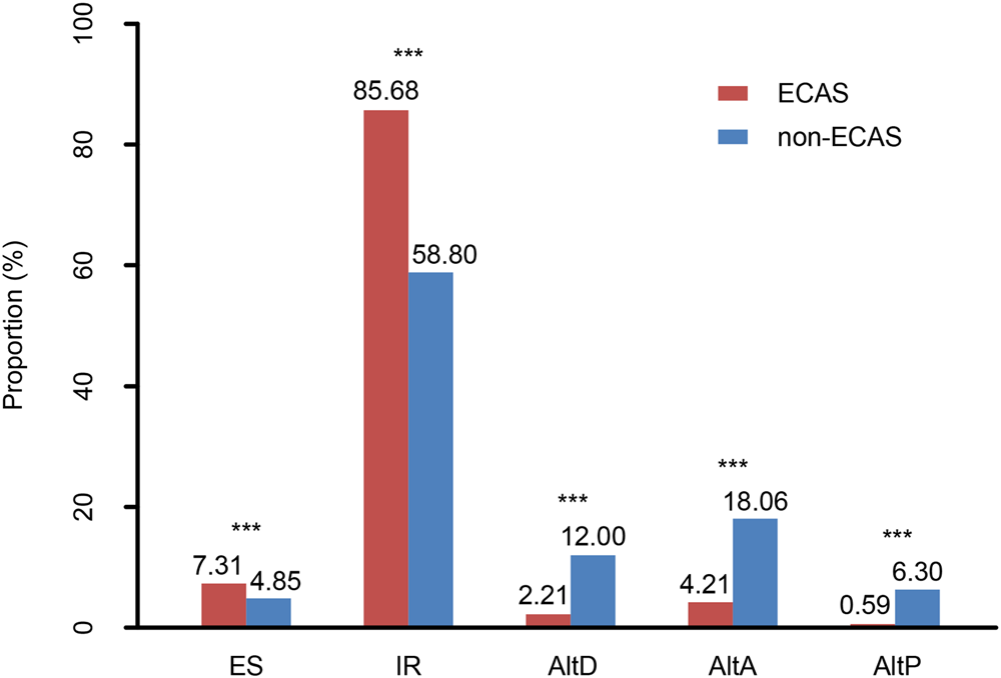
Distribution of alternative splicing (AS) types in evolutionarily conserved AS (ECAS) events and non-ECAS events. ***P < 0.001. ES, exon skipping; IR, intron retention; AltD, alternative donor; AltA, alternative acceptor; AltP, alternative position.

We analyzed the genic features of ECAS events in comparison with non-ECAS events in *Sonneratia* species. Among the five AS types, IR and ES events were the two most enriched types in ECAS events (85.68% and 7.31%, respectively, Fig. 4; Supplementary Table S4), followed by AltA (4.21%) and alternative donor (AltD, 2.21%). In contrast, IR and AltA events were the most common types among non-ECAS events (58.80% and 18.06%, respectively). Moreover, IR and ES events in the ECAS events were significantly overrepresented compared with those in the non-ECAS events (Wilcoxon test, *P* < 0.001 for both classes), but the three other types AltD, AltA and AltP were significantly less abundant (*P* < 0.001 for all the three classes).

For ECAS events, the average length of the retained intron and skipped exon was 122.96 and 90.56 bp, respectively. The deleted and added fragments involved in AltD and AltA events had a short average length of 57.83 and 29.63 bp, respectively (Table 2). Interestingly, the lengths of all four AS types among the ECAS events were shorter than those in the corresponding types of non-ECAS events, three of which (IR, ES and AltA) were of significance (Wilcoxon test, *P* < 0.001, 0.05 and 0.01 for the three classes, respectively). With regard to the location of AS events, we found no significant difference in each type of AS event between ECAS and non-ECAS events (Supplementary Table S5). Most ECAS and non-ECAS events occurred in the coding regions (85.02% and 84.66%, respectively; G-test, *P* = 0.781), followed by 3′UTR (8.08% and 7.87%, respectively; *P* = 0.833) and 5′UTR regions (6.90% and 7.47%, respectively; *P* = 0.544).

**Table 2:** Length of alternative splicing (AS) types for evolutionarily conserved AS (ECAS) events and non-ECAS events (bp).

	ES	IR	AltD	AltA
**ECAS**				
Mean	90.56	122.96	57.83	29.63
Median	73	96	24.5	5
**non-ECAS**				
Mean	119.00	161.05	131.11	74.88
Median	80	114	30	12
**Wilcoxon test**				
*P*-value	0.029	<2.2*10^−16 ***^	0.332	0.004

ES, exon skipping; IR, intron retention; AltD, alternative donor; AltA, alternative acceptor.

^***^P < 0.001.

A total of 719 (83.80%) and 2167 (87.03%) premature stop codons (PTCs) were identified from 858 ECAS events and 2490 non-ECAS events, respectively (Table 3). Of them, significantly higher proportion of IR isoforms with PTCs were identified in non-ECAS events than in ECAS events (G-test, *P* < 0.001). Correspondingly, non-ECAS events were more likely to lead to frameshifts than the ECAS events, according to the total number of frameshifts introduced by AS events (Table 4). Overall, 115 frameshifts (13.40%) were found in the conserved events, which was significantly less than the 622 (24.98%) found in the non-ECAS events (G-test, *P* < 0.001). Among the five AS types, AltA events exhibited significantly less frameshifts in the ECAS events compared with the non-ECAS events (G-test, *P* < 0.05). These results suggest that ECAS events may have more functional conservation than non-ECAS events.

**Table 3:** Statistics of premature termination codons (PTCs) introduced by evolutionarily conserved AS (ECAS) events and non-ECAS events.

	ES	IR	AltD	AltA	AltP	Total
**ECAS**						
No. of PTCs (%)	7 (70.00)	146 (81.56)	2 (100.00)	2 (25.00)	1 (50.00)	158 (78.61)
No. of non-PTCs (%)	3 (30.00)	33 (18.44)	0 (0.00)	6 (75.00)	1 (50.00)	43 (21.39)
**non-ECAS**						
No. of PTCs (%)	89 (93.68)	1312 (92.39)	189 (82.53)	270 (68.70)	68 (81.93)	1928 (86.85)
No. of non-PTCs (%)	6 (6.32)	108 (7.61)	39 (17.03)	122 (31.04)	15 (18.07)	290 (13.06)
**G-test (on ECAS vs non-ECAS)**						
*P*-value	0.035^*^	1.4*10^−5 ***^	0.388	0.012^*^	0.314	0.002^**^

ES, exon skipping; IR, intron retention; AltD, alternative donor; AltA, alternative acceptor; AltP, alternative position; No., number.

^*^P < 0.05; ^**^P < 0.01; ^***^P < 0.001.

Only genes meeting the three criteria described in Methods were taken into account.

**Table 4:** Statistics of frameshifts introduced by evolutionarily conserved AS (ECAS) events and non-ECAS events.

	ES	IR	AltD	AltA	AltP	Total
**ECAS**						
No. of frameshifts (%)	2 (20.00)	17 (9.50)	1 (50.00)	0 (0.00)	1 (50.00)	21 (10.45)
No. of non-frameshifts (%)	8 (80.00)	162 (90.50)	1 (50.00)	8 (100.00)	1 (50.00)	180 (89.55)
**non-ECAS**						
No. of frameshifts (%)	46 (48.42)	114 (8.03)	146 (63.76)	192 (48.85)	58 (69.88)	556 (25.05)
No. of non-frameshifts (%)	49 (51.58)	1306 (91.97)	83 (36.24)	201 (51.15)	25 (30.12)	1664 (74.95)
**G-test (on ECAS vs non-ECAS)**						
*P*-value	0.075	0.508	0.693	0.001^*^	0.563	4.6*10^−7 ***^

ES, exon skipping; IR, intron retention; AltD, alternative donor; AltA, alternative acceptor; AltP, alternative position; No., number.

^*^P < 0.05; ^***^P < 0.001.

Only genes meeting the three criteria described in Methods were taken into account.

We found that three protein domains from three genes were modified by ES events, while three domains from two genes were lost due to ES events (Supplementary Table S6). Two other domains from two genes were modified by AltA events. 10 and 69 domains on 10 and 48 genes were modified and lost due to IR events, respectively, while four new domains were introduced by intron retention in four genes.

### Functional annotation of genes with the evolutionarily conserved alternative splicing (ECAS) events

GO classification was performed for the 1,170 genes with ECAS events for functional annotation. We found 26 GO terms with significant overrepresentation at the third GO level (*P* < 0.05, Supplementary Table S7). Of them, six, nine and 11 terms belonged to the categories of cell component, molecular function and biological process. In the category of molecular function, four of the nine enriched terms were annotated as proton-transporting ATPase activity (GO: 0046961), zinc ion binding (GO: 0008270) and calcium ion binding (GO: 0005509), which may play a critical role in the adaptation to environmental stresses. In the two terms ATP hydrolysis coupled proton transport (GO: 0015991) and proton-transporting ATPase activity, rotational mechanism (GO: 0046961, Supplementary Table S8), two genes (SA_12345 and SA_28678) encode two vacuolar ATP synthase (V-ATPase) subunit A family proteins, while two other genes (SA_07404 and SA_18019) encode V-ATPase F family proteins. The product of another gene (SA_14841) is homologous to vacuolar H^+^-ATPase subunit E isoform 1 (VHA-E1). In another GO term calcium ion binding (GO: 0005509), genes SA_04445, SA_26340, SA_06082 and SA_12609 encode alpha-amylase-like protein (AMY1), two phosphoinositide-specific phospholipase C family proteins, PLC2 and PLC15, and salt overly sensitive 3 (SOS3), respectively.

Interestingly, 60 genes with ECAS events were annotated with functions of response to stress (GO: 0006950) and abiotic stress (GO: 0009628), although no significant overabundance was detected for these two GO terms. Of them, 15 genes were directly involved in the response to osmotic stress (GO: 0006970), including salt stress and water deprivation (Supplementary Table S9). Four genes, SA_21670, SA_22710, SA_24912 and SA_00745, encode heat-shock protein 90.7 (HSP90.7), CBL-interacting protein kinase 9 (CIPK9), arm repeat protein interacting with ABF2 (ARIA) and a cold-inducible peroxidase Rare Cold Inducible gene 3 (RCI3), respectively. 11 genes were identified with functions related to oxidative and heat stress resistance (GO: 0006979 and GO: 0009408, Supplementary Table S9). Three of them (SA_07415, SA_07247 and SA_16454) were annotated as microsomal glutathione S-transferase, glutamate-cysteine ligase (GCL) and ascorbate peroxidase 1 (APX1), which are involved in the metabolic processes of two important antioxidant metabolites, glutathione and ascorbate.

### Evolutionarily conserved alternative splicing (ECAS) validation by Reverse transcription-PCR (RT-PCR)

**Figure 5:**
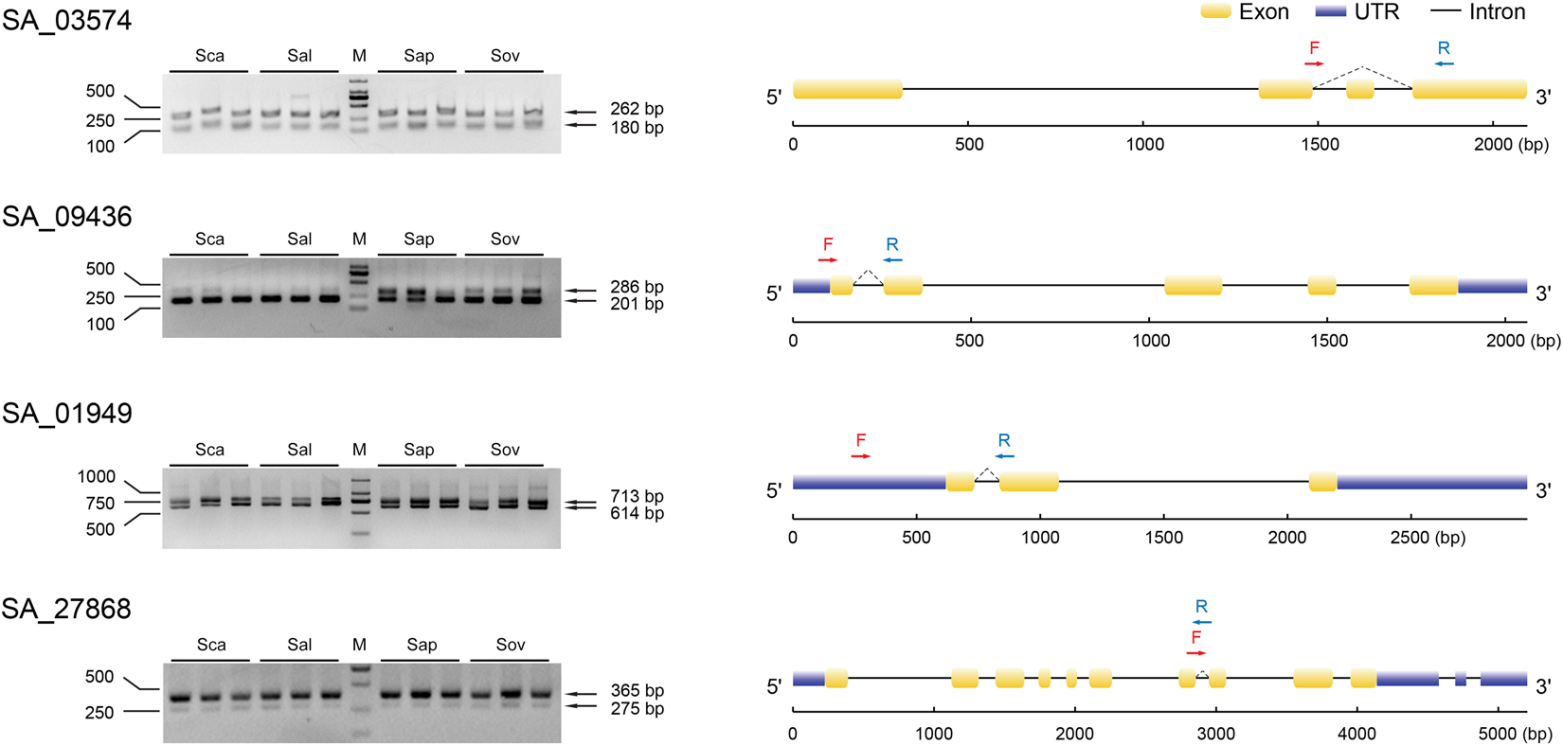
Reverse transcription-PCR (RT-PCR) validations for four evolutionarily conserved alternative splicing (ECAS) events in four *Sonneratia* species. Splicing events are represented by dash lines connecting exons. The positions and the sizes of different isoforms are labeled by arrows and numbers right to the arrows. Sca, *S. caseolaris*; Sal, *S. alba*; Sap, *S. apetala*; Sov, *S. ovata*; M, DNA ladders. Gene architecture for each gene was constructed using Gene Structure Display Server (GSDS) v. 2.0^69^. Primer sets used for splicing event validation were highlighted in red (F, forward) and blue (R, reverse).

To validate the accuracy of our identified evolutionary conserved alternative splicing events in four *Sonneratia* species, 11 alternatively spliced genes were randomly selected for reverse transcription PCR (RT-PCR). The results showed that all the 11 genes had two isoforms corresponding to constitutive and alternative spliced isoforms, respectively, in each of the four species (Fig. 5; Supplementary Fig. S4), although some isoforms were of low expression level. This suggested a reliable detection of ECAS events in the *Sonneratia* species.

## Discussion

According to our results, 77,351 to 103,127 SJs were identified in 14,298–15,787 genes in the four *Sonneratia* species, 88.04 to 93.14% of which were located in CDS regions (Supplementary Fig. S1). A similar observation has also been found in other species, thus highlighting the potential of splice junctions to affect final proteins and their functions^2^. Overall, we identified 7,248 to 12,623 AS events from 25.16% to 34.62% expressed genes in four *Sonneratia* species (Supplementary Table S1), and the percentages were close to those in *Vitis vinifera* (30%)^32^ and *P. trichocarpa* (36%)^6^. The proportions of alternatively spliced genes in *Sonneratia* were lower than those in maize (40%)^7^, rice (48%)^8^, *Arabidopsis* (61%)^2^ and soybean (63%)^9^ identified from multiple lines, tissues or developmental stages by next-generation sequencing. Previous studies have revealed that some AS events occur at specific developmental stages, in tissues, cells or in response to environmental stress conditions, including both abiotic and biotic stimulus^1,2,33,34^. In the present study, the number of AS events in the four *Sonneratia* species was estimated through root transcriptome sequencing under the simulated normal growth conditions in the greenhouse, which may have led to an underestimation of AS abundance. Despite this, the frequencies of the five major AS types in all four *Sonneratia* species were similar to other terrestrial plant species^2,6,35^. IR events were the most overrepresented (63.82–73.22%, Fig. 1), matching previous findings in plants^36^.

Generally, AS events can be influenced by many internal factors, such as hormone-response elements, chromatin modifications and splice site usage^37–39^. Moreover, several studies have shown that gene structure, including intron number, intron length, and transcriptional level dramatically alter AS frequency^9,40^. In contrast, a study in *Arabidopsis* showed no significant correlation between the proportion of IR events and intron size^2^. In this study, we revealed a significant correlation between AS frequency and gene features in *Sonneratia*, in agreement with previous observations in soybean^9^. Our analysis showed that AS frequency was significantly concomitant with an increase in gene transcriptional levels in three of the four species (Fig. 2a). Furthermore, the occurrence of IR and ES events positively correlated with intron or exon numbers but negatively correlated with intron or exon length in all four *Sonneratia* species (Fig. 2b,c; Supplementary Figs S2 and S3). Although AS regulation is complex and requires further investigations, our results provide clues regarding the major factors involved in the control of AS.

Identification of ECAS events is thought to be a good indicator of the functional significance of alternative splicing genes^3,4,36^. For the ECAS events between different species pairs, the highest level of conservation was between *S. ovata* and *S. apetala* (35.71%), followed by between *S. alba* and *S. ovata* (34.79%), and between *S. alba* and *S. apetala* (24.94%, Supplementary Table S2). In contrast, *S. caseolaris* had a relatively low level of ECAS with the three other species. The varying conservation level between different species pairs of *Sonneratia* was not conjugated with the inter-specific sequence divergence, unlike the observation in *Drosophila*^17^. It may be due to the underestimation of AS events in the four *Sonneratia* species by using transcriptome data from only roots, since lots of AS events have been identified as tissue-specific in both mammals and plants^1,41,42^.

For all four *Sonneratia* species, 1,355 ECAS events were detected from 1,170 genes (Supplementary Table S3). Of them, approximately 86% of these events occurred in the coding region (Supplementary Table S5), consistent with the proportion observed in previous studies^4,14^. IR and ES were more overrepresented in ECAS events than in non-ECAS events (*P* < 0.01, Fig. 4; Supplementary Table S4). This result is inconsistent with the observation between *Brassica* and *Arabidopsis*, in which these two types were less abundant in ECAS events than non-ECAS events^14^. This conflict implies that the conservation patterns of AS may be different in different lineages of plants or may differ at the intrageneric and intergeneric levels. The first explanation seems more probable because the conservation patterns should be similar between intrageneric and intergeneric levels under neutral evolution. With regard to conserved IR and ES events, the length of exons and introns was significantly shorter than that in non-conserved events (Table 2). ECAS isoforms with shorter length are thought to have less influence on protein sequence and stability, which is unlikely to be selected against or to be functionally important^14^.

Similarly, the introduction of PTCs by IR was significantly less likely in ECAS events than in non-ECAS events (Table 3). Empirical views indicated that IR within the coding regions may highly affect RNA posttranscriptional metabolism by introducing PTCs, which are targets for mRNA degeneration through NMD^10–12^. According to our results, ECAS events in *Sonneratia* have less deleterious impacts on gene products than non-ECAS events, suggesting greater functional significance of the ECAS events.

GO classification revealed the functional information of the genes presenting ECAS events among the four species of *Sonneratia* (Supplementary Table S7). For the six genes assigned to the GO term ATP hydrolysis coupled proton transport (GO: 0015991, Supplementary Table S8), the gene SA_07404 encoding ATPase, F1 complex, alpha subunit protein has been reported to be one of “salt and osmotic stress responsive proteins”^43^. Another three genes were annotated as the members of *V-ATPase* family. V-ATPase has been reported to play an important role in tolerance to environmental stress in plants^44^. Of them, SA_14841 encodes a protein VHA-E1, which plays a key role in the functional secretory system during embryonic development^45^. In the GO term calcium ion binding (GO: 0005509), one gene (SA_04445) is the homolog of *AMY1* gene in *Arabidopsis*. In *Arabidopsis*, the transcription of AMY1 is induced upon exposure to heat or salt stress, suggesting a functional role in abiotic-stress tolerance^46,47^. PLC2 and PLC15, which are encoded by SA_26340 and SA_06082, respectively, have been implicated in the phosphatidylinositol (PI) signaling pathway, which is critical in the plant responses to heat, drought and salt stresses^48^. Moreover, Qin *et al*. found that *PLC15* could be induced by the overexpression of *ZmDREB2A* in maize when seedlings faced such stresses^49^. Another gene, SA_12609, encodes a protein with an important function in salt tolerance in plants, SOS3. Generally, SOS3 is thought to be a sensor of the changes in the concentration of intracellular calcium^50^. Under salt stress conditions, SOS3 forms the SOS3-SOS2 complex with SOS2, and then activates the downstream plasma membrane-localized Na^+^/H^+^ antiporter SOS1, which protect plants from the damage of toxic Na^+^^51^. The ECAS events in these stress-response genes may be essential for specific adaptations to intertidal habitats in *Sonneratia*. In another GO term, oxidative phosphorylation (GO: 0006119), the gene (SA_26130) encoding vacuolar H^+^-pyrophosphatase 2 (VHP2) has similar function of Na^+^ translocation to vacuoles in Arabidopsis, rice and bread wheat (*Triticum aestivum*)^52^. Another proton ATPase protein plasma membrane proton ATPase (PMA), encoded by SA_07445, can promote salt tolerance by improving K^+^ influx^53^. PMA has also been reported to participate in maintaining the carbon balance in response to heat stress in *Arabidopsis*^54^. The ECAS events in these ion transport-relative genes suggest that they might play a potential regulatory role in the adaptation of *Sonneratia* to high-saline intertidal zones.

In addition to these overrepresented GO terms, other three GO terms for genes with the ECAS events were relevant to the response to osmotic, oxidative and heat stresses (Supplementary Table S9). In the term response to osmotic stress (GO: 0006970), 15 genes were directly involved in the tolerance to salt stress and water deprivation in plants. Of them, the gene encoding Hsp90.7 protein (SA_21670) is relevant to the resistance to salt and drought stresses in plants via regulating the ABA-dependent or Ca^2+^ signal pathways^55^. Moreover, two proteins, ARIA and RCI3, are also involved in the ABA and salt stress responses^56,57^. ARIA positively regulates ABA response during the germination stage by interacting with ABF2 or NIMA-related kinase 6 (NEM6), and enhances the salt tolerance during the subsequent stage of seedling growth^56,58^. Another protein, CIPK9, is required to maintain ionic equilibrium under abiotic stress conditions^59^.

In the last two categories, 11 genes were assigned to functions of oxidative and heat stress resistance. Glutamate-cysteine ligase (GCL), the product of the gene SA_07415, catalyzes the first step of glutathione biosynthesis from L-cysteine and L-glutamate to gamma-glutamylcysteine^60^. Glutathione is an essential antioxidant metabolite for the prevention of the ROS damage to cellular components under drought and high salt stress conditions. Furthermore, together with ascorbate, glutathione also functions in the ROS detoxification pathway ascorbate - glutathione (AsA - GSH) cycle in plants^61^. In this study, we also identified a gene (SA_07247) that encodes the ascorbate peroxidase 1 (APX1) with an ECAS event in *Sonneratia*, suggesting that it may also play a crucial role in *Sonneratia* in the adaptation to harsh intertidal habitats. In summary, ECAS events in the stress-tolerance related genes identified here may provide new insights into the molecular mechanisms underlying salt tolerance in *Sonneratia*, and also offer an important resource for further functional investigations.

In conclusion, our findings suggested that ECAS events are of functional importance and implicated in adaptation to abiotic stresses in *Sonneratia* species, which provides new insights into post-transcriptional regulatory mechanisms of mangrove plants in response to stressful marine environments. Our study also provides important clues for further functional and evolutionary studies in mangrove plants.

## Methods

### Plant materials and transcriptome sequencing

The transcriptome of *S. alba* was sequenced previously^24^ and transcriptomes of three other species of *Sonneratia* were sequenced in this study. Seedlings of *S. caseolaris*, *S. ovata* and *S. apetala* were collected from Wenchang, Hainan, China and cultivated in a greenhouse. After the seedlings returned to normal growth, root tissues were harvested from each species and quickly frozen in liquid nitrogen for RNA isolation. We used only root tissues for comparison because, (1) only roots were used for transcriptome sequencing in *S. alba* previously^24^ and (2) AS are often organ-specific^32^. Total RNA was extracted using the modified CTAB method^62^. Paired-end libraries were constructed for each species using an Illumina mRNA-Seq Prep Kit and sequenced on the Illumina Genome Analyzer (*S. caseolaris*) and HiSeq. 2000 (*S. ovata* and *S. apetala*) platforms (Illumina Inc.). After trimming the adaptor sequence from the raw sequence data, low-quality reads, which had an average base quality of less than 20, had a base quality of 20 for 20% bases or more, or had 5% ambiguous bases (N bases) or more, were removed from each dataset.

### Alignment of the reads to the S. alba reference genome

*S. alba* genome sequences were downloaded from EMBL database (accession number PRJEB8424). Filtered reads from each sample were aligned to the *S. alba* reference genome using the BLAT program^63^. For *S. alba*, the criterion for high-quality alignments was ≥96% sequence identity. To minimize false mapping and select reliable cross species read alignments, the identity cutoff was set to 92% for *S. caseolaris* and to 94% for *S. ovata* and *S. apetala* according to the sequence divergence level between each species and *S. alba*^23^. For the reads with multiple hits, the hit with the highest identity was chosen as the optimal hit. The RPKM (reads per kilobase of exon model per million mapped reads) values were computed and normalized using the Enhanced Read Analysis of Gene Expression (ERANGE) strategy as described by Mortazavi *et al*.^64^. For genes with more than one gene models in the genome annotation, the longest one was selected for the calculation.

### Splice junction (SJ) detection

Splicing patterns were explored using the Read Analysis & Comparison Kit in Java (RACKJ) software toolbox (http://rackj.sourceforge.net/), which separately calculates the number of reads matched to exons, introns and splice junctions^40,65,66^. To diminish the number of potential false positives that are predicted by erroneous alignment, we required a junction site to be supported by at least two reads per ten million totally mapped reads, with all supporting reads having a minimum of eight bases on both side of the junction and at least two of them having a non-repetitive match position.

We defined those junctions that are within the coordinates of annotated genes in the *S. alba* genome as genic splice junctions. Those junctions beyond any gene coordinates were called intergenic junctions. For the genic splice junctions, we examined whether the predicted splice junctions were inside the coding sequence (CDS), 5′ UTR or 3′ UTR of protein-coding genes. If one end of the junction was in the coding sequence and the other was in the UTR they were classified as 5′UTR-CDS or CDS-3′UTR, as described in Marquez *et al*^2^.

### Detection of alternative splicing (AS) events

We investigated the five main types of alternative splicing in plants: exon skipping (ES), intron retention (IR), alternative donor (AltD), alternative acceptor (AltA), and alternative position (AltP, in which both donor and acceptor sites are different). Based on the mapping results, RACKJ was used to compute the following read counts, and the results were separated into several Tables: (1) for each exon, (2) for each intron, (3) for each exon-pair that was mapped by spliced reads, and (4) for each exon-pair plus junction shifts that were supported by spliced reads. AS events were then computed accordingly. The fourth table records potential AltA/AltD/AltP events. If there was evidence of a 5′ splice site (SS) spliced to multiple 3′SS, this event was classified as an AltD. If a 3′SS was spliced to multiple 5′SS, this event was classified as an AltA. An AltP event refers to two splice junctions affecting the same exon pair but different 5′SS and 3′SS. To determine IR, we used the following thresholds: a minimum of two intron-mapped reads per ten million totally mapped reads, and a minimum of 0.5× coverage across the mapped intron.

To explore the influence of genic features and gene transcriptional level on AS, we calculated the correlations of three comparisons for all genes between the AS distribution and gene expression level, between the IR distribution and each of intron number and the length of retained intron, and between the ES distribution and each of exon number and the length of skipped exon. The statistics of correlation R^2^ and *P*-value were calculated with the R package (v. 3.13, http://cran.r-project.org/).

### Identifying evolutionarily conserved alternative splicing (ECAS) events in the four species of *Sonneratia*

Here evolutionarily conserved AS (ECAS) event refers to the same AS event shared by at least two species of *Sonneratia*. To identify ECAS events, the types and genomic coordinates of the AS events predicted from the four species were compared (1) between any two of the four species, (2) among any three of the four species, and (3) among all four species. Conserved ES/IR events were identified as the same type of events leading to the skipping/retention of homologous exons/introns in the orthologous genes between species. Conserved AltD/AltA/AltP events were defined as the same type of events affecting the same exon-intron junctions and having the same position in the orthologous genes between species. If there was evidence for one or more ECAS event (as described above) in a gene, this gene was characterized as an ECAS gene. Other events that show no evidence for conservation (found in only one species) were classified as non-ECAS events.

We compared the distributions, lengths, positions, and effects on introduction of premature stop codons (PTCs) and frameshifts between ECAS and non-ECAS events. The distributions and length of AS events for each AS type were calculated for all the identified ECAS events and non-ECAS events. Wilcoxon tests and G-tests were used to determine whether there were significant differences between them, respectively. The length of IR and ES events were defined as the length of the retained intron or skipped exon, and the length of AltD and AltA events represented the number of base pairs deleted or added at one end of an intron. To compare the position of AS events, genes containing only one AS event were analyzed and the locations of AS events in the coding region, 5′ UTR and 3′ UTR were identified. To calculate the number of PTCs and frameshifts introduced by ECAS and non-ECAS events, only the alternatively spliced genes meeting each of the following conditions were taken into account: (1) genes contain only one AS event; (2) the AS events is located in the coding region; and (3) the start codon is not affected by the AS event. The significant difference between ECAS and non-ECAS events was tested by the G-test.

We further examined the impacts of ECAS events on the protein domains by comparing the domains generated by constitutive and alternative isoforms. Protein domains were identified using HMMER 3.1^67^ with a cutoff of e-value < 1E-10. Only genes that contain only one AS event and whose start codon is not affected by the AS event, were taken into account. If at least one domain is completely lost, we consider it as a domain loss, and if a new domain is introduced by an AS event, we consider it as a domain gain. If a part of domain is modified by an AS event, we consider it as a domain modification. To infer the functional role of these ECAS genes in *Sonneratia*, Gene Ontology (GO) enrichment analysis was performed with the topGO software^68^.

### Reverse transcription-PCR (RT-PCR) validation for alternatively spliced isoforms

To validate the ECAS events identified in four *Sonneratia* species, 11 genes with ECAS events were randomly selected for Reverse transcription-PCR (RT-PCR). For each species, root tissues were collected from three individuals as biological replicates. 0.5 µg of RNA was reverse transcribed using ReverTra Ace qPCR RT Kit 101 (Toyobo, Osaka, Japan), and PCR was performed in a 50 μL reaction mixture containing 1 μL of KOD FX polymerase (Toyobo, Osaka, Japan), 25 μL of the 2× PCR buffer supplied by the manufacturer, and 10 μL of 2 mM dNTPs. The PCR conditions were 4 min at 94 °C, followed by 30 cycles of 10 s at 98 °C, 30 s at 60 °C, 7 min at 68 °C, and a final extension of 10 min at 68 °C in a Bio-Rad S1000 (Bio-Rad Laboratories, CA, USA). All the primer sequences used in this study were listed in Supplementary Table S10. The amplified fragments were detected by electrophoresis. Gene architecture of four of the 11 genes, SA_03574, SA_09436, SA_01949 and SA_27868, were constructed using Gene Structure Display Server (GSDS) v. 2.0^69^.

### Data availability

Raw reads of the four transcriptomes have been submitted to the Sequence Read Archive of NCBI with accession numbers SRP068401 (*S. alba*: SRS1246730; *S. caseolaris*: SRS1246739; *S. apetala*: SRS1246741; *S. ovata*: SRS1246742).

## Electronic supplementary material

Supplementary information
