# Supplementary material for: Identification and characterization of evolutionarily conserved alternative splicing events in a mangrove genus Sonneratia
Source: Sci Rep. 2018 Mar 13;8:4425. doi: 10.1038/s41598-018-22406-6 (PMC5849712; doi:10.1038/s41598-018-22406-6)
Supplement: Supplementary file 1 — Supplementary information [file 41598_2018_22406_MOESM1_ESM.pdf]

SUPPLEMENTARY INFORMATION

Identification and characterization of evolutionarily conserved alternative splicing events in a mangrove genus *Sonneratia*

Yuchen Yang[1,2], Wuxia Guo[1], Xu Shen[1], Jianfang Li[1], Shuhuan Yang[1], Sufang Chen[1], Ziwen He[1], Renchao Zhou[1*] and Suhua Shi[1*]

[1]State Key Laboratory of Biocontrol and Guangdong Provincial Key Laboratory of Plant Resources, Sun Yat-sen University, Guangzhou 510275, China. [2]Department of Genetics, Department of Biostatistics, Department of Computer Science University of North Carolina, Chapel Hill, NC 27599, U.S.A.

Supplementary Figure S1. Distribution of splicing junction (SJ) positions in protein-coding genes in four *Sonneratia* species.

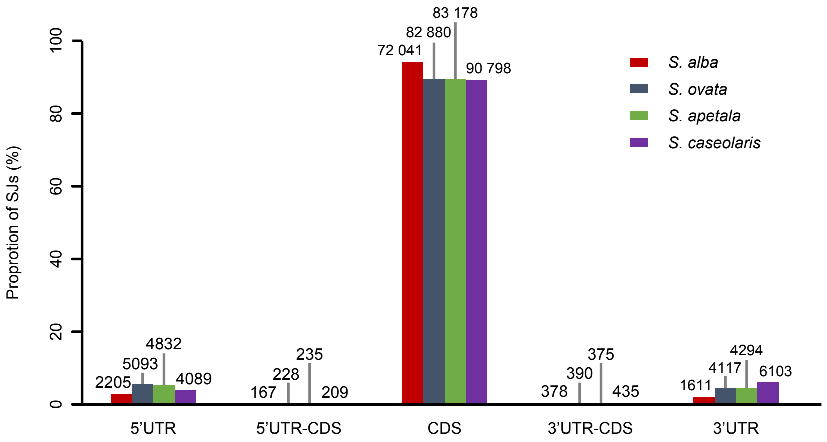

Supplementary Figure S2. Correlations between the proportion of exon skipping (ES) events and exon numbers, as well as exon length, of genes in four *Sonneratia* species. (a) Correlations between the proportion of ES events and the exon number per gene in four *Sonneratia* species. **(b)** Correlations between the proportion of ES events and the exon length of genes in four *Sonneratia* species.

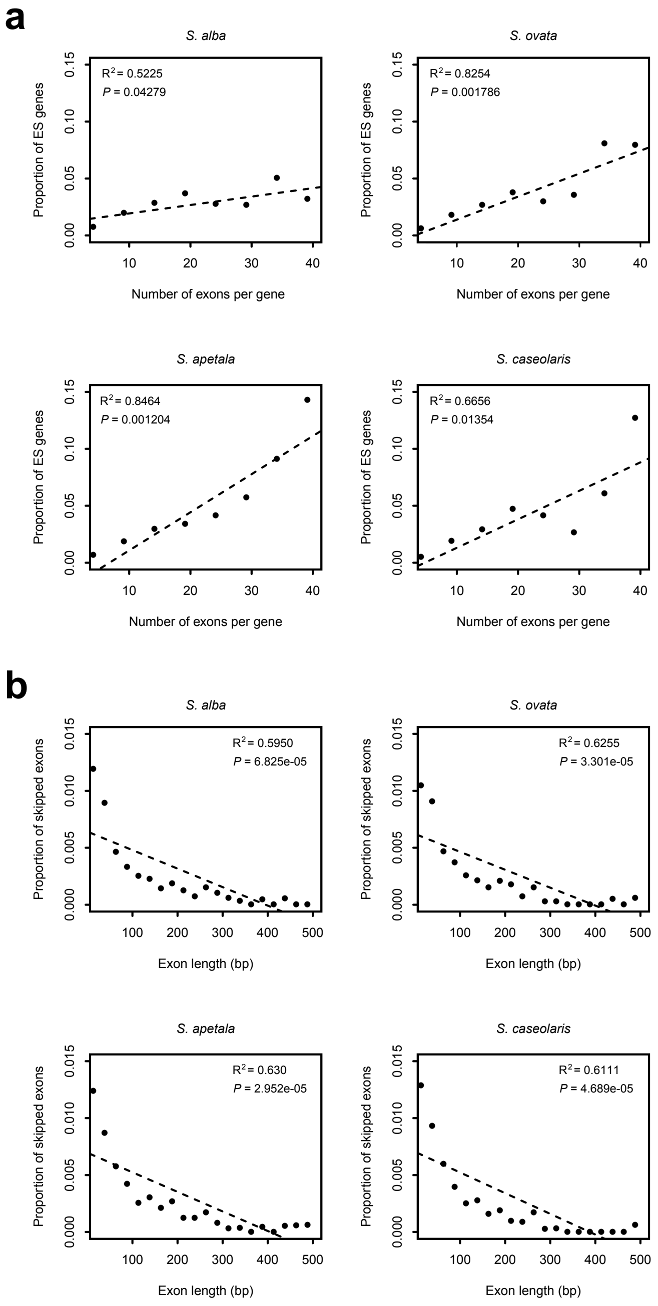

Supplementary Figure S3. Correlation between the frequency of evolutionarily conserved alternative splicing (ECAS) events and inter-specific sequence divergence in species pairs of *Sonneratia*.

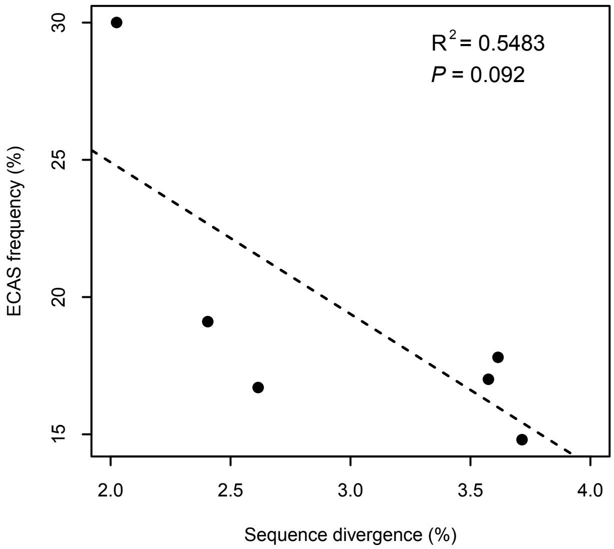

Supplementary Figure S4. Reverse transcription-PCR (RT-PCR) validations for 11 evolutionarily conserved alternative splicing (ECAS) events in four *Sonneratia* species. The positions and the sizes of different isoforms are labeled by arrows and numbers right to the arrows. Sca, S. caseolaris; Sal, S. alba; Sap, S. apetala; Sov, S. ovata; M, DNA ladders.

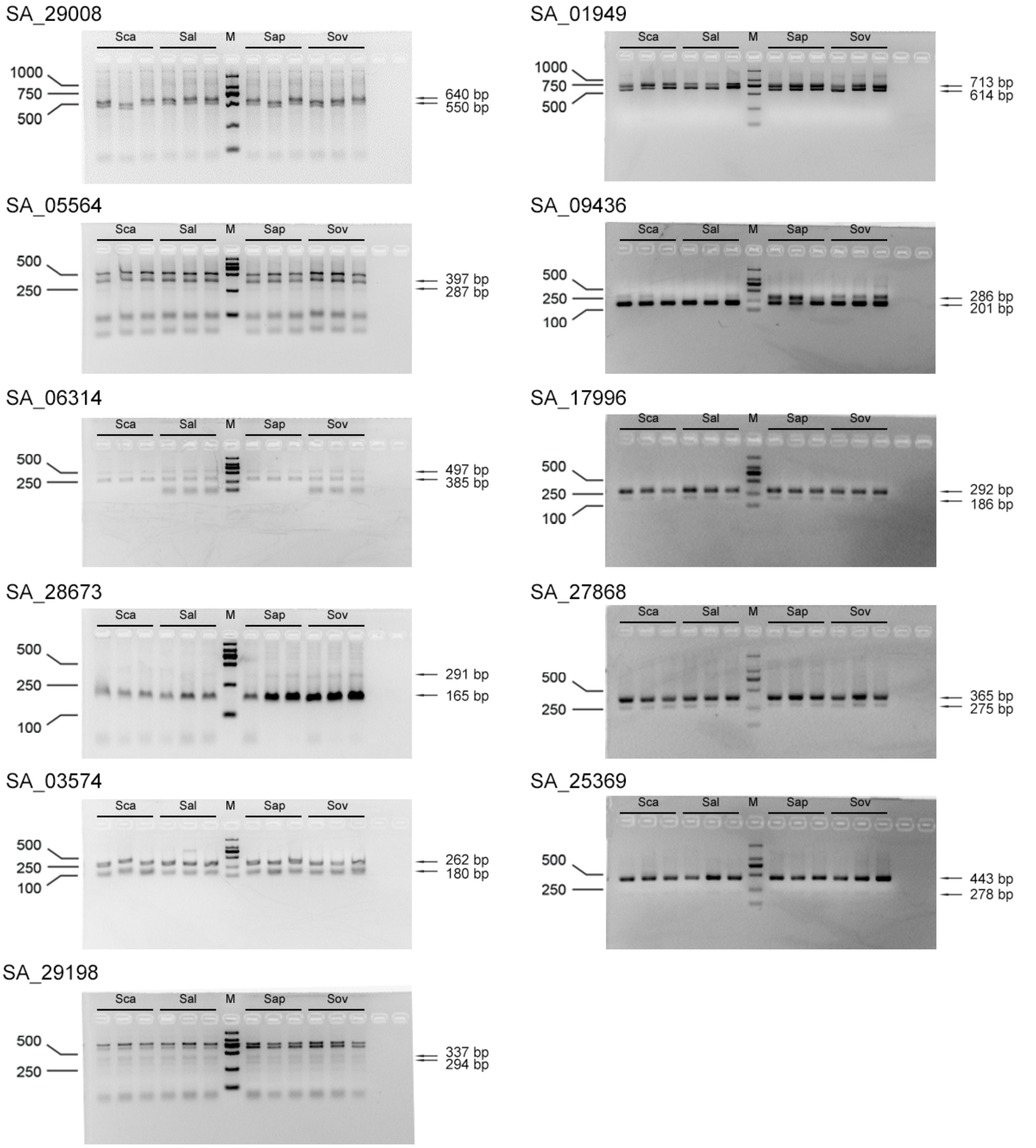

Supplementary Table S1. Distributions of alternative splicing (AS) types in four *Sonneratia* species.

	ES	IR	AltD	AltA	AltP	Total
S. alba						
Events (%)	385 (5.31)	4626 (63.82)	737 (10.17)	1121 (15.47)	379 (5.23)	7248
Genes (%)	374 (7.79)	3500 (72.86)	665 (13.84)	994 (20.69)	299 (6.22)	4804 (25.16)
S. ovata						
Events (%)	361 (3.87)	6823 (73.22)	438 (4.70)	775 (8.32)	921 (9.88)	9318
Genes (%)	352 (6.19)	4608 (81.06)	401 (7.05)	721 (12.68)	768 (13.51)	5685 (28.40)
S. apetala						
Events (%)	399 (3.38)	8532 (72.29)	563 (4.77)	895 (7.58)	1414 (11.98)	11 803
Genes (%)	387 (6.06)	5245 (82.13)	514 (8.05)	807 (12.64)	1103 (17.27)	6386 (33.24)
S. caseolaris						
Events (%)	382 (3.03)	8168 (64.71)	665 (5.27)	1251 (9.91)	2157 (17.09)	12 623
Genes (%)	368 (5.33)	5349 (77.47)	622 (9.01)	1114 (16.13)	1730 (25.05)	6905 (34.62)

ES, exon skipping; IR, intron retention; AltD, alternative donor; AltA, alternative acceptor; AltP, alternative position.

Supplementary Table S2. Distributions of alternative splicing (AS) types for evolutionarily conserved AS (ECAS) events between two species and among three species of *Sonneratia*.

	ES	IR	AltD	AltA	AltP	Total
ECAS events between two species (%)						
S. alba - S. ovata	163 (6.13)	2196 (82.56)	92 (3.46)	186 (6.99)	23 (0.86)	2660 (19.13)
S. alba - S. apetala	170 (6.25)	2232 (82.09)	99 (3.64)	196 (7.21)	22 (0.81)	2719 (16.65)
S. alba - S. caseolaris	159 (6.20)	2078 (81.01)	103 (4.02)	205 (7.99)	20 (0.78)	2565 (14.82)
S. ovata - S. apetala	226 (4.63)	3938 (80.76)	177 (3.63)	317 (6.50)	218 (4.47)	4876 (30.02)
S. ovata - S. caseolaris	172 (5.41)	2654 (83.43)	82 (2.58)	202 (6.35)	71 (2.23)	3181 (16.96)
S. apetala - S. caseolaris	179 (4.84)	3075 (83.22)	113 (3.06)	233 (6.31)	95 (2.57)	3695 (17.82)
ECAS events among three species (%)						
S. alba - S. ovata - S. apetala	134 (6.80)	1652 (83.77)	60 (3.04)	113 (5.73)	13 (0.66)	1972 (9.82)
S. alba - S. ovata - S. caseolaris	112 (6.82)	1398 (85.14)	41 (2.50)	82 (4.99)	9 (0.55)	1642 (7.32)
S. alba - S. apetala - S. caseolaris	117 (6.92)	1427 (84.44)	47 (2.78)	88 (5.21)	11 (0.65)	1690 (6.93)
S. ovata - S. apetala - S. caseolaris	140 (5.91)	1998 (84.38)	57 (2.41)	128 (5.41)	45 (1.90)	2368 (9.72)
ECAS events among four species (%)						
S. alba - *S. ovata - S. apetala - S. caseolaris*	99 (7.31)	1161 (85.68)	30 (2.21)	57 (4.21)	8 (0.59)	1355 (4.91)

ES, exon skipping; IR, intron retention; AltD, alternative donor; AltA, alternative acceptor; AltP, alternative position.

Supplementary Table S3. Gene ID, locations and descriptions of the genes containing evolutionarily conserved alternative splicing (ECAS) events among four *Sonneratia* species.

Gene ID	Scaffold ID	Exon Pair/No. of Retained Intron/Splice	Gene description
Exon skipping			
SA_01474	scaffold_1102	5<=>7	Serine/threonine-protein phosphatase 5
SA_01999	scaffold_1102	1<=>3	Auxin-binding protein T85, putative
SA_02033	scaffold_1102	1<=>4	
SA_02220	scaffold_1102	2<=>4	Ubiquitin-fold modifier 1, putative
SA_02405	scaffold_1132	2<=>4	Putative uncharacterized protein
SA_02614	scaffold_1132	2<=>4	Putative uncharacterized protein
SA_02727	scaffold_1132	6<=>8	Receptor protein kinase, putative
SA_02780	scaffold_1132	1<=>3	Bel1 homeotic protein, putative
SA_02804	scaffold_1132	3<=>5	Protein kinase family protein
SA_02947	scaffold_1132	1<=>3	Putative uncharacterized protein
SA_03417	scaffold_1153	2<=>4	Glucan endo-1,3-beta-glucosidase, putative
SA_03431	scaffold_1153	4<=>23	Pyrophosphate-fructose 6-phosphate 1-phosphotransferase subunit beta
SA_03574	scaffold_1281	2<=>4	Pollen-specific protein
SA_03951	scaffold_1391	1<=>3	Cbs domain protein
SA_04145	scaffold_1391	1<=>3	Pyruvate dehydrogenase alpha subunit
SA_04404	scaffold_142	3<=>5	Putative uncharacterized protein
SA_04599	scaffold_144	1<=>3	Glucuronokinase 1
SA_04867	scaffold_1463	2<=>4	Breast carcinoma amplified sequence, putative
SA_04877	scaffold_1463	3<=>5	B12D-like protein
SA_06327	scaffold_24	29<=>31	Transferase, transferring glycosyl groups, putative
SA_07247	scaffold_244	2<=>4	Glutamate--cysteine ligase, chloroplast, putative
SA_07445	scaffold_265	1<=>3	H(\+)-transporting atpase plant/fungi plasma membrane type, putative
SA_08196	scaffold_301	2<=>4	PLE, putative
SA_08221	scaffold_301	4<=>6	Suppressor of ty, putative
SA_08447	scaffold_301	1<=>3	Nucleosome/chromatin assembly factor group
SA_09060	scaffold_333	6<=>8	U1 small nuclear ribonucleoprotein 70 kDa, putative
SA_09170	scaffold_333	13<=>15	Cytosolic purine 5-nucleotidase, putative
SA_09290	scaffold_333	5<=>8	Inter-alpha-trypsin inhibitor heavy chain, putative
SA_11035	scaffold_388	3<=>5	ATP binding protein, putative
SA_11060	scaffold_388	8<=>10	Ubiquitin-like modifier-activating enzyme
SA_11211	scaffold_394	4<=>6	Ubiquitin-conjugating enzyme E2-like protein
SA_11724	scaffold_396	1<=>3	Strubbelig receptor family 3
SA_12122	scaffold_400	4<=>6	Predicted protein
SA_12668	scaffold_407	1<=>3	Fasciclin-like AGP 12
SA_12692	scaffold_407	10<=>12	Putative uncharacterized protein

SA_12743	scaffold_407	1<=>3	Putative uncharacterized protein
SA_12798	scaffold_407	2<=>4	Leucine-rich repeat protein, putative
SA_12828	scaffold_407	3<=>5	Mitochondrial fission 1 protein
SA_13185	scaffold_667	2<=>4	Ring finger protein, putative
SA_13531	scaffold_705	12<=>14	Binding protein
SA_13729	scaffold_705	2<=>5	Cysteine proteinase inhibitor
SA_13961	scaffold_705	5<=>7	Or protein
SA_14620	scaffold_795	8<=>10	Ecotropic viral integration site, putative
SA_14833	scaffold_891	1<=>4	
SA_14851	scaffold_891	4<=>6	40S ribosomal protein S10, putative
SA_14968	scaffold_891	6<=>8	15-hydroxyprostaglandin dehydrogenase
SA_16025	scaffold_897	8<=>10	Putative uncharacterized protein
SA_16518	scaffold_899	6<=>8	Putative uncharacterized protein
SA_16715	scaffold_899	2<=>4	Light-inducible protein CPRF2
SA_16733	scaffold_899	4<=>6	Predicted protein (Fragment)
SA_16790	scaffold_899	4<=>6	UDP-glucuronic acid decarboxylase 2
SA_16981	scaffold_899	1<=>3	Calmodulin binding protein, putative
SA_17896	scaffold_916	5<=>8	Transcription factor, putative
SA_18019	scaffold_916	1<=>4	V-type proton ATPase subunit F
SA_18455	scaffold_917	8<=>10	Predicted protein
SA_18660	scaffold_917	4<=>7	Potassium transporter, putative
SA_18745	scaffold_917	5<=>7	Uncharacterized protein
SA_19167	scaffold_918	11<=>13	Lactoylglutathione lyase
SA_19202	scaffold_918	12<=>14	Putative uncharacterized protein
SA_19313	scaffold_918	3<=>5	Ubiquitin-like protein
SA_19781	scaffold_918	5<=>7	Putative uncharacterized protein
SA_20121	scaffold_918	13<=>15	Uncharacterized protein
SA_20831	scaffold_922	2<=>4	Putative uncharacterized protein
SA_21107	scaffold_922	12<=>14	Predicted protein
SA_21241	scaffold_922	6<=>8	Thioredoxin I, putative
SA_21470	scaffold_923	2<=>4	ATP binding protein, putative
SA_21477	scaffold_923	1<=>4	Scythe/bat3, putative
SA_21520	scaffold_923	13<=>15	CBL-interacting serine/threonine-protein kinase, putative
SA_21926	scaffold_923	3<=>5	Putative uncharacterized protein
SA_22856	scaffold_927	9<=>11	Putative uncharacterized protein
SA_23502	scaffold_929	2<=>4	60S acidic ribosomal protein P1, putative
SA_23661	scaffold_929	5<=>7	Protein YME1, putative
SA_23697	scaffold_929	4<=>6	Predicted protein
SA_23919	scaffold_929	2<=>4	Putative uncharacterized protein
SA_24491	scaffold_930	1<=>3	Putative uncharacterized protein
SA_24804	scaffold_930	2<=>5	Anamorsin homolog
SA_24824	scaffold_930	6<=>8	Putative uncharacterized protein

SA_25246	scaffold_930	11<=>13	Eukaryotic translation initiation factor 3 subunit E
SA_25436	scaffold_930	1<=>3	Cysteine-type peptidase
SA_25692	scaffold_934	9<=>11	PERK1-like protein kinase
SA_25902	scaffold_934	7<=>9	Pollen-specific protein SF3, putative
SA_26168	scaffold_942	5<=>7	Uncharacterized protein
SA_26169	scaffold_942	7<=>9	MIKC-type MADS-box transcription factor WM30
SA_26531	scaffold_942	10<=>12	Cleavage and polyadenylation specificity factor, putative
SA_26719	scaffold_942	2<=>4	3-ketoacyl CoA thiolase 1
SA_26770	scaffold_942	1<=>3	Glycine rich protein-like
SA_27464	scaffold_948	11<=>13	Putative uncharacterized protein
SA_27561	scaffold_948	5<=>7	14-3-3-like protein (Fragment)
SA_27721	scaffold_948	1<=>3	Ubiquitin family protein
SA_27827	scaffold_954	5<=>7	Predicted protein
SA_28081	scaffold_954	3<=>5	Putative uncharacterized protein
SA_28196	scaffold_954	4<=>6	
SA_28280	scaffold_954	3<=>5	Cytochrome b reductase
SA_28303	scaffold_954	4<=>6	Chaperone binding protein, putative
SA_28443	scaffold_957	1<=>3	Predicted protein (Fragment)
SA_28455	scaffold_957	3<=>5	Cytochrome C oxidase polypeptide vib, putative
SA_28673	scaffold_983	1<=>3	Putative uncharacterized protein
SA_29084	scaffold_995	5<=>7	Protein AINTEGUMENTA, putative
SA_29198	scaffold_995	1<=>3	Calmodulin 2
Intron retention			
SA_00118	scaffold_1017	1	Putative uncharacterized protein
SA_00210	scaffold_1017	3	Hypoxanthine-guanine phosphoribosyltransferase, putative
SA_00231	scaffold_1017	12	Translation initiation factor if-2, putative
SA_00267	scaffold_1017	5	AarF domain-containing protein kinase, putative
SA_00402	scaffold_1030	1	UDP-glucose:glycoprotein glucosyltransferase
SA_00402	scaffold_1030	2	UDP-glucose:glycoprotein glucosyltransferase
SA_00403	scaffold_1030	1	Serine hydroxymethyltransferase
SA_00403	scaffold_1030	2	Serine hydroxymethyltransferase
SA_00412	scaffold_1030	2	At4g24330
SA_00448	scaffold_1030	8	Fuct c3 protein
SA_00510	scaffold_1030	5	Putative uncharacterized protein
SA_00511	scaffold_1030	1	At3g09980
SA_00550	scaffold_1030	8	Putative uncharacterized protein
SA_00555	scaffold_1030	14	Rhomboid protein, putative
SA_00570	scaffold_1030	2	Uncharacterized protein
SA_00570	scaffold_1030	3	Uncharacterized protein
SA_00600	scaffold_1030	1	TGA-type basic leucine zipper protein TGA2.2
SA_00627	scaffold_1030	6	GTPase activating protein
SA_00745	scaffold_1030	1	Peroxidase 3, putative

SA_00765	scaffold_1030	1	Armadillo/beta-catenin repeat family protein
SA_00849	scaffold_1030	14	Beta-1,3-galactosyltransferase sqv-2, putative
SA_00878	scaffold_1030	7	Transcriptional corepressor SEUSS, putative
SA_00878	scaffold_1030	9	Transcriptional corepressor SEUSS, putative
SA_00896	scaffold_1030	1	Putative uncharacterized protein
SA_00903	scaffold_1050	11	PMP, putative
SA_00910	scaffold_1050	8	Structural maintenance of chromosome 1 protein, putative
SA_00915	scaffold_1050	2	Protein UNUSUAL FLORAL ORGANS, putative
SA_00928	scaffold_1050	1	Trithorax, putative
SA_00930	scaffold_1050	5	Ubiquitin
SA_00932	scaffold_1050	3	Aspartic proteinase nepenthesin-1, putative
SA_00982	scaffold_1050	6	SOC1-like protein
SA_01008	scaffold_1096	4	DNA-binding protein
SA_01018	scaffold_1096	2	Starch synthase isoform II
SA_01028	scaffold_1096	8	Pentatricopeptide repeat-containing protein At3g53700, chloroplastic
SA_01032	scaffold_1096	2	mRNA, clone: RTFL01-47-M12
SA_01085	scaffold_1096	6	Putative uncharacterized protein
SA_01146	scaffold_1096	1	UDP-glucosyltransferase, putative
SA_01150	scaffold_1096	13	Amino acid binding protein, putative
SA_01152	scaffold_1096	3	Molybdopterin-binding, putative
SA_01186	scaffold_1096	3	Run and tbc1 domain containing 3, plant, putative
SA_01197	scaffold_1096	1	UDP-glucuronic acid decarboxylase 1
SA_01203	scaffold_1096	4	Acetylglucosaminyltransferase, putative
SA_01261	scaffold_1096	8	Putative uncharacterized protein
SA_01279	scaffold_1096	13	Putative uncharacterized protein
SA_01302	scaffold_1096	13	N-acetyl-glutamate synthase
SA_01302	scaffold_1096	3	N-acetyl-glutamate synthase
SA_01351	scaffold_1098	3	Uncharacterized protein
SA_01394	scaffold_1102	17	Allantoinase, putative
SA_01447	scaffold_1102	10	Amine oxidase, putative
SA_01469	scaffold_1102	2	Predicted protein
SA_01518	scaffold_1102	5	Putative uncharacterized protein
SA_01518	scaffold_1102	6	Putative uncharacterized protein
SA_01526	scaffold_1102	1	Peroxisomal membrane protein, putative
SA_01532	scaffold_1102	10	F-box family protein
SA_01532	scaffold_1102	7	F-box family protein
SA_01605	scaffold_1102	3	Predicted protein
SA_01605	scaffold_1102	5	Predicted protein
SA_01612	scaffold_1102	2	Acyl-CoA thioesterase, putative
SA_01631	scaffold_1102	1	GTPase-activating protein
SA_01670	scaffold_1102	1	Ferric reductase
SA_01714	scaffold_1102	5	Potassium transporter, putative

SA_01737	scaffold_1102	2	Predicted protein
SA_01764	scaffold_1102	1	Adenosine diphosphatase, putative
SA_01770	scaffold_1102	6	Ribulose-1,5 bisphosphate carboxylase/oxygenase large subunit N-methyltransferase, chloroplast, putative
SA_01794	scaffold_1102	11	Serine/threonine protein kinase, putative
SA_01799	scaffold_1102	7	Translin-associated protein X
SA_01816	scaffold_1102	5	ATP binding protein, putative
SA_01816	scaffold_1102	6	ATP binding protein, putative
SA_01894	scaffold_1102	1	Putative uncharacterized protein
SA_01949	scaffold_1102	1	Predicted protein
SA_01970	scaffold_1102	1	Cytochrome P450, putative
SA_01989	scaffold_1102	1	Serine endopeptidase degp2, putative
SA_01989	scaffold_1102	2	Serine endopeptidase degp2, putative
SA_02025	scaffold_1102	5	CRS2-associated factor 2, mitochondrial
SA_02046	scaffold_1102	1	Catalytic, putative
SA_02156	scaffold_1102	7	GATA domain class transcription factor
SA_02185	scaffold_1102	12	Putative uncharacterized protein
SA_02224	scaffold_1102	2	Nuclear inhibitor of protein phosphatase-1, putative
SA_02240	scaffold_1102	2	Nucleic acid binding protein, putative
SA_02264	scaffold_1102	15	Retinoblastoma-binding protein, putative
SA_02264	scaffold_1102	16	Retinoblastoma-binding protein, putative
SA_02269	scaffold_1102	4	Protein-tyrosine phosphatase mitochondrial
SA_02301	scaffold_1102	7	Tobamovirus multiplication 2B
SA_02332	scaffold_1102	6	Protein bem46, putative
SA_02339	scaffold_1102	6	Probable methyltransferase PMT13
SA_02342	scaffold_1102	1	
SA_02345	scaffold_1102	2	Methylenetetrahydrofolate dehydrogenase, putative
SA_02352	scaffold_1102	2	Protein AFR, putative
SA_02352	scaffold_1102	3	Protein AFR, putative
SA_02354	scaffold_1102	10	Beta-amylase 6
SA_02360	scaffold_1102	1	Putative uncharacterized protein
SA_02373	scaffold_1102	1	Putative uncharacterized protein
SA_02402	scaffold_1132	3	Nuclear transcription factor Y subunit C-1
SA_02408	scaffold_1132	4	AMP dependent CoA ligase, putative
SA_02420	scaffold_1132	4	Afc, putative
SA_02420	scaffold_1132	5	Afc, putative
SA_02509	scaffold_1132	2	
SA_02521	scaffold_1132	1	Glycine-rich RNA-binding protein, putative
SA_02560	scaffold_1132	2	Haloacid dehalogenase-like hydrolase
SA_02570	scaffold_1132	1	
SA_02575	scaffold_1132	1	Glycogenin, putative
SA_02576	scaffold_1132	1	Metalloprotease m41 ftsh, putative
SA_02598	scaffold_1132	7	BZIP transcription factor

SA_02621	scaffold_1132	12	GAUT7/LGT7 (Fragment)
SA_02694	scaffold_1132	5	Putative uncharacterized protein
SA_02721	scaffold_1132	6	Serine/threonine-protein kinase cx32, putative
SA_02780	scaffold_1132	5	Bel1 homeotic protein, putative
SA_02780	scaffold_1132	6	Bel1 homeotic protein, putative
SA_02797	scaffold_1132	1	Response to dessication RD2
SA_02847	scaffold_1132	1	
SA_02861	scaffold_1132	6	Chloroplast vanilla cream 1
SA_02861	scaffold_1132	8	Chloroplast vanilla cream 1
SA_02862	scaffold_1132	8	Putative uncharacterized protein
SA_02887	scaffold_1132	1	Uncharacterized protein
SA_02889	scaffold_1132	5	Putative uncharacterized protein
SA_02940	scaffold_1132	2	Uncharacterized protein
SA_02961	scaffold_1132	7	Predicted protein (Fragment)
SA_02999	scaffold_1132	8	Cholinephosphate cytidylyltransferase, putative
SA_03062	scaffold_1132	4	Predicted protein
SA_03107	scaffold_1132	1	Putative uncharacterized protein
SA_03107	scaffold_1132	4	Putative uncharacterized protein
SA_03124	scaffold_1132	1	Global transcription factor group
SA_03137	scaffold_1132	1	Uncharacterized protein
SA_03218	scaffold_1145	11	Developmentally-regulated GTP-binding protein 2
SA_03279	scaffold_1153	8	Golgi SNAP receptor complex member 1
SA_03293	scaffold_1153	27	Putative uncharacterized protein
SA_03365	scaffold_1153	1	At1g61340
SA_03370	scaffold_1153	7	Structural molecule, putative
SA_03402	scaffold_1153	20	Adenosine deaminase, putative
SA_03402	scaffold_1153	21	Adenosine deaminase, putative
SA_03426	scaffold_1153	1	Predicted protein
SA_03465	scaffold_1153	10	WD-repeat protein, putative
SA_03465	scaffold_1153	11	WD-repeat protein, putative
SA_03465	scaffold_1153	9	WD-repeat protein, putative
SA_03473	scaffold_1153	8	GTP-binding protein erg, putative
SA_03473	scaffold_1153	9	GTP-binding protein erg, putative
SA_03521	scaffold_1153	1	Glycosyltransferase QUASIMODO1, putative
SA_03539	scaffold_122	4	50S ribosomal protein L12, chloroplastic
SA_03546	scaffold_122	7	Potassium transporter, putative
SA_03571	scaffold_1281	1	
SA_03620	scaffold_1281	1	BTB/POZ domain-containing protein
SA_03731	scaffold_1290	2	Cinnamyl alcohol dehydrogenase
SA_03745	scaffold_1290	1	Uncharacterized protein
SA_03770	scaffold_1290	1	
SA_03774	scaffold_1290	6	RNA 3' terminal phosphate cyclase, putative
SA_03774	scaffold_1290	7	RNA 3' terminal phosphate cyclase, putative

SA_03778	scaffold_1290	23	Putative uncharacterized protein
SA_03809	scaffold_1290	9	RNA polymerase II ctd phosphatase, putative
SA_03817	scaffold_1290	6	Uncharacterized protein
SA_03833	scaffold_1290	1	Senescence-associated protein
SA_03841	scaffold_1290	3	Predicted protein (Fragment)
SA_03842	scaffold_1290	6	Protein translocase secy subunit, putative
SA_03890	scaffold_1290	1	Protein phosphatase 2a, regulatory subunit, putative
SA_03893	scaffold_1290	1	DVL10
SA_03933	scaffold_1290	1	40S ribosomal protein S30
SA_03971	scaffold_1391	1	Putative uncharacterized protein
SA_03978	scaffold_1391	10	Putative uncharacterized protein
SA_03978	scaffold_1391	9	Putative uncharacterized protein
SA_04019	scaffold_1391	3	GRAS family protein (Fragment)
SA_04024	scaffold_1391	2	Cell wall protein Exp5
SA_04070	scaffold_1391	19	Protein binding protein, putative
SA_04110	scaffold_1391	8	Ganglioside induced differentiation associated protein, putative
SA_04157	scaffold_1391	1	
SA_04158	scaffold_1391	9	Amsh, putative
SA_04288	scaffold_142	1	Putative uncharacterized protein
SA_04308	scaffold_142	4	Oligopeptide transporter, putative
SA_04343	scaffold_142	2	AP-1 complex subunit gamma-2, putative
SA_04343	scaffold_142	3	AP-1 complex subunit gamma-2, putative
SA_04372	scaffold_142	2	Thioredoxin
SA_04376	scaffold_142	1	Ubiquitin conjugating enzyme
SA_04388	scaffold_142	3	Rer1 protein, putative
SA_04391	scaffold_142	17	Sugar transporter, putative
SA_04392	scaffold_142	1	Lrr receptor protein kinase, putative
SA_04409	scaffold_142	1	Rnf5, putative
SA_04415	scaffold_142	7	Putative uncharacterized protein
SA_04445	scaffold_142	14	Alpha-amylase
SA_04552	scaffold_144	18	Acylamino-acid-releasing enzyme, putative
SA_04566	scaffold_144	6	At3g01370
SA_04586	scaffold_144	2	BC10 protein
SA_04719	scaffold_1463	2	Putative uncharacterized protein
SA_04719	scaffold_1463	8	Putative uncharacterized protein
SA_04724	scaffold_1463	2	Pentatricopeptide repeat-containing protein
SA_04724	scaffold_1463	3	Pentatricopeptide repeat-containing protein
SA_04747	scaffold_1463	1	Kinase, putative
SA_04759	scaffold_1463	2	50S ribosomal protein L17, chloroplastic
SA_04764	scaffold_1463	1	Glycosyl transferase, family 8
SA_04886	scaffold_1463	22	Mannosyl-oligosaccharide 1,2-alpha-mannosidase IA, putative
SA_04909	scaffold_1463	5	Protein kinase atn1, putative

SA_04909	scaffold_1463	6	Protein kinase atn1, putative
SA_04920	scaffold_1463	8	Putative uncharacterized protein
SA_04921	scaffold_1463	4	Putative uncharacterized protein
SA_04931	scaffold_1463	1	1-deoxy-D-xylulose 5-phosphate reductoisomerase
SA_04944	scaffold_1463	1	Predicted protein (Fragment)
SA_04970	scaffold_1463	3	Pentatricopeptide repeat-containing protein, putative
SA_04994	scaffold_1463	7	Uncharacterized protein
SA_05083	scaffold_1463	10	Heat-shock protein 105 kDa, putative
SA_05088	scaffold_1463	9	Calcium-dependent protein kinase, putative
SA_05103	scaffold_1463	11	Ubiquitin-activating enzyme E1, putative
SA_05129	scaffold_1463	14	Chromatin regulatory protein sir2, putative
SA_05138	scaffold_1463	1	AGAP006959-PA
SA_05163	scaffold_152	14	Multidrug/pheromone exporter, MDR family, ABC transporter family
SA_05163	scaffold_152	2	Multidrug/pheromone exporter, MDR family, ABC transporter family
SA_05172	scaffold_152	2	Heat shock 70kDa protein 1/8
SA_05201	scaffold_165	11	Cullin-1, putative
SA_05201	scaffold_165	12	Cullin-1, putative
SA_05201	scaffold_165	7	Cullin-1, putative
SA_05281	scaffold_165	1	Transcription factor, putative
SA_05304	scaffold_165	1	Putative uncharacterized protein
SA_05316	scaffold_165	4	Multidrug resistance protein ABC transporter family
SA_05318	scaffold_165	11	Ubiquitin-protein ligase, putative
SA_05347	scaffold_165	1	ATOZI1, putative
SA_05365	scaffold_165	16	Sensor histidine kinase, putative
SA_05530	scaffold_219	14	Nucleosome assembly protein, putative
SA_05535	scaffold_219	1	Mitochondrial carrier protein RIM2, putative
SA_05564	scaffold_219	1	Predicted protein
SA_05587	scaffold_219	7	RAC-like small GTPase
SA_05616	scaffold_219	4	Inorganic pyrophosphatase, putative
SA_05702	scaffold_219	5	Aspartyl-tRNA synthetase
SA_05749	scaffold_219	1	Uncharacterized protein
SA_05799	scaffold_219	6	At1g20890
SA_05829	scaffold_219	5	Uncharacterized protein
SA_05849	scaffold_219	11	Ein3-binding f-box protein 3
SA_05892	scaffold_219	3	
SA_05896	scaffold_219	1	Putative uncharacterized protein
SA_05912	scaffold_219	1	Uncharacterized protein
SA_05982	scaffold_221	4	Protein TRF-like 8
SA_05995	scaffold_221	6	Protein containing C-terminal RING-finger
SA_06082	scaffold_221	3	1-phosphatidylinositol-4,5-bisphosphate phosphodiesterase, putative
SA_06115	scaffold_221	9	Uncharacterized protein

SA_06157	scaffold_231	1	Putative uncharacterized protein
SA_06200	scaffold_24	2	
SA_06202	scaffold_24	1	Chromodomain-helicase-DNA-binding protein
SA_06224	scaffold_24	12	Predicted protein
SA_06227	scaffold_24	4	Predicted protein
SA_06254	scaffold_24	12	Ubiquitin carboxyl-terminal hydrolase
SA_06258	scaffold_24	10	Cellular nucleic acid-binding protein
SA_06314	scaffold_24	1	Putative uncharacterized protein
SA_06345	scaffold_24	1	At1g11880
SA_06374	scaffold_24	30	ATP binding protein, putative
SA_06380	scaffold_24	7	Transaldolase, putative
SA_06383	scaffold_24	21	Putative uncharacterized protein
SA_06383	scaffold_24	27	Putative uncharacterized protein
SA_06383	scaffold_24	9	Putative uncharacterized protein
SA_06433	scaffold_24	11	Regulator of G-protein signaling 1
SA_06437	scaffold_24	8	Dentin sialophosphoprotein, putative
SA_06444	scaffold_24	11	Uncharacterized protein
SA_06513	scaffold_24	1	Protein kinase APK1B, chloroplast, putative
SA_06513	scaffold_24	2	Protein kinase APK1B, chloroplast, putative
SA_06523	scaffold_24	6	MYB domain class transcription factor
SA_06551	scaffold_24	21	Ganglioside induced differentiation associated protein, putative
SA_06562	scaffold_24	12	ATP-dependent RNA helicase, putative
SA_06623	scaffold_24	4	AP2/ERF domain-containing transcription factor
SA_06682	scaffold_24	1	Tubulin gamma complex-associated protein
SA_06687	scaffold_24	1	Glucosamine-fructose-6-phosphate aminotransferase, putative
SA_06736	scaffold_24	1	
SA_06774	scaffold_24	2	Protein binding protein, putative
SA_06793	scaffold_24	13	DNA-directed RNA polymerase, putative
SA_06846	scaffold_24	19	Peroxisome biogenesis factor, putative
SA_06849	scaffold_24	3	Chitinase (Precursor)
SA_06849	scaffold_24	8	Chitinase (Precursor)
SA_06870	scaffold_24	3	Ubiquitin-conjugating enzyme 32
SA_06870	scaffold_24	4	Ubiquitin-conjugating enzyme 32
SA_06881	scaffold_24	6	Chaperone protein dnaJ
SA_06920	scaffold_244	3	Inner membrane protein
SA_06954	scaffold_244	2	Uncharacterized protein
SA_07011	scaffold_244	11	Predicted protein
SA_07065	scaffold_244	13	Uncharacterized protein
SA_07119	scaffold_244	12	Peroxidase
SA_07169	scaffold_244	10	N-acetyl-gamma-glutamyl-phosphate reductase
SA_07203	scaffold_244	9	ATP-dependent Clp protease proteolytic subunit

SA_07210	scaffold_244	2	Leucine-rich repeat transmembrane protein kinase, putative
SA_07213	scaffold_244	1	
SA_07221	scaffold_244	12	Putative uncharacterized protein
SA_07257	scaffold_244	18	Peptidase M1 family protein
SA_07299	scaffold_244	3	N-rich protein, putative
SA_07300	scaffold_244	1	Cell division protein ftsH, putative
SA_07328	scaffold_244	3	Predicted protein
SA_07339	scaffold_244	14	Serine/threonine protein kinase, putative
SA_07339	scaffold_244	7	Serine/threonine protein kinase, putative
SA_07372	scaffold_244	3	Component of oligomeric golgi complex, putative
SA_07372	scaffold_244	7	Component of oligomeric golgi complex, putative
SA_07374	scaffold_244	8	Uncharacterized protein
SA_07404	scaffold_260	1	ATP synthase subunit alpha, mitochondrial
SA_07404	scaffold_260	2	ATP synthase subunit alpha, mitochondrial
SA_07404	scaffold_260	3	ATP synthase subunit alpha, mitochondrial
SA_07415	scaffold_265	11	Microsomal glutathione s-transferase, putative
SA_07477	scaffold_265	12	Catalytic, putative
SA_07507	scaffold_265	2	Amino acid carrier
SA_07511	scaffold_265	15	Protein disulfide isomerase, putative
SA_07595	scaffold_267	12	Transcription factor HBP-1b(C1), putative
SA_07635	scaffold_267	4	Alpha-xylosidase, putative
SA_07693	scaffold_267	3	Alpha-crystallin domain of heat shock protein-containing protein
SA_07700	scaffold_267	17	Mitogen activated protein kinase kinase kinase 3, mapkkk3, mekk3, putative
SA_07708	scaffold_267	3	Predicted protein
SA_07727	scaffold_267	3	Pectinesterase
SA_07766	scaffold_267	5	Nucleic acid binding protein, putative
SA_07766	scaffold_267	6	Nucleic acid binding protein, putative
SA_07772	scaffold_267	6	F10A16.24 protein
SA_07773	scaffold_267	9	Dead box ATP-dependent RNA helicase, putative
SA_07775	scaffold_267	9	
SA_07788	scaffold_270	1	COL domain class transcription factor
SA_07813	scaffold_270	12	Nucleobase ascorbate transporter
SA_07843	scaffold_270	5	WRKY transcription factor, putative
SA_07878	scaffold_270	12	Uncharacterized protein
SA_07909	scaffold_270	5	Thylakoid membrane phosphoprotein 14 kDa, chloroplast, putative
SA_07918	scaffold_270	1	ATP binding protein, putative
SA_08008	scaffold_282	4	Predicted protein
SA_08038	scaffold_282	2	Chorismate mutase
SA_08049	scaffold_282	4	UDP-glucuronosyltransferase, putative
SA_08051	scaffold_282	2	Protein binding protein, putative
SA_08079	scaffold_282	12	Putative uncharacterized protein

SA_08083	scaffold_282	4	Predicted protein
SA_08118	scaffold_282	10	Putative uncharacterized protein
SA_08118	scaffold_282	5	Putative uncharacterized protein
SA_08125	scaffold_297	8	Putative uncharacterized protein
SA_08146	scaffold_297	3	Putative uncharacterized protein
SA_08154	scaffold_301	1	
SA_08157	scaffold_301	3	Expressed protein
SA_08208	scaffold_301	2	ATP-dependent Clp protease proteolytic subunit, putative
SA_08229	scaffold_301	1	ATP-dependent RNA helicase, putative
SA_08298	scaffold_301	9	Initiation factor eIF-4 gamma, middle; Up-frameshift suppressor 2
SA_08352	scaffold_301	2	Predicted protein
SA_08354	scaffold_301	4	At5g05950
SA_08368	scaffold_301	4	Microsomal signal peptidase 25 kD subunit, putative
SA_08378	scaffold_301	6	
SA_08442	scaffold_301	17	Hect ubiquitin-protein ligase, putative
SA_08446	scaffold_301	13	Predicted protein
SA_08468	scaffold_301	12	Predicted protein
SA_08468	scaffold_301	4	Predicted protein
SA_08501	scaffold_301	1	Aspartokinase
SA_08544	scaffold_301	1	Protein phosphatase 2A regulatory subunit B' eta
SA_08544	scaffold_301	3	Protein phosphatase 2A regulatory subunit B' eta
SA_08544	scaffold_301	4	Protein phosphatase 2A regulatory subunit B' eta
SA_08644	scaffold_301	3	Predicted protein
SA_08658	scaffold_301	3	Ethylene-responsive element-binding protein
SA_08698	scaffold_330	1	Tyrosine phosphatase 1
SA_08736	scaffold_330	1	Lipid binding protein, putative
SA_08753	scaffold_330	1	Pentatricopeptide repeat-containing protein, putative
SA_08836	scaffold_330	20	Uncharacterized protein
SA_08894	scaffold_330	1	Putative uncharacterized protein
SA_08894	scaffold_330	2	Putative uncharacterized protein
SA_08906	scaffold_333	1	Putative uncharacterized protein
SA_08910	scaffold_333	2	Putative uncharacterized protein
SA_08955	scaffold_333	11	Malate dehydrogenase
SA_08999	scaffold_333	1	Heat shock protein 70 (HSP70)-interacting protein, putative
SA_08999	scaffold_333	2	Heat shock protein 70 (HSP70)-interacting protein, putative
SA_09034	scaffold_333	4	Protein SSM1, putative
SA_09034	scaffold_333	7	Protein SSM1, putative
SA_09068	scaffold_333	1	
SA_09091	scaffold_333	5	Phosphoric diester hydrolase, putative
SA_09124	scaffold_333	11	Importin subunit alpha
SA_09124	scaffold_333	8	Importin subunit alpha

SA_09124	scaffold_333	9	Importin subunit alpha
SA_09135	scaffold_333	10	Map3k delta-1 protein kinase, putative
SA_09226	scaffold_333	1	Heat shock protein binding protein, putative
SA_09286	scaffold_333	1	
SA_09286	scaffold_333	3	
SA_09303	scaffold_333	15	Phosphoinositide 5-phosphatase, putative
SA_09400	scaffold_333	1	Putative uncharacterized protein
SA_09436	scaffold_333	1	ADP-ribosylation factor, putative
SA_09472	scaffold_333	3	Fructose-6-phosphate 2-kinase/fructose-2,6-bisphosphatase
SA_09486	scaffold_333	1	Putative uncharacterized protein
SA_09486	scaffold_333	10	Putative uncharacterized protein
SA_09486	scaffold_333	12	Putative uncharacterized protein
SA_09499	scaffold_333	1	Predicted protein
SA_09508	scaffold_333	2	Predicted protein
SA_09509	scaffold_333	6	Pentatricopeptide repeat-containing protein
SA_09544	scaffold_333	1	Putative uncharacterized protein
SA_09575	scaffold_333	14	AP-2 complex subunit alpha, putative
SA_09627	scaffold_342	1	F-box and wd40 domain protein, putative
SA_09785	scaffold_346	15	Ankyrin repeat domain protein, putative
SA_09835	scaffold_346	13	Minichromosome maintenance protein 10
SA_09835	scaffold_346	14	Minichromosome maintenance protein 10
SA_09837	scaffold_346	13	Dead box ATP-dependent RNA helicase, putative
SA_09858	scaffold_346	12	Protein COBRA, putative
SA_09858	scaffold_346	9	Protein COBRA, putative
SA_09866	scaffold_346	5	Aldo-keto reductase, putative
SA_09870	scaffold_346	7	Expressed protein
SA_09918	scaffold_350	6	Putative uncharacterized protein
SA_09964	scaffold_352	8	Putative uncharacterized protein
SA_09998	scaffold_364	3	Alpha-L-fucosidase 2, putative
SA_10025	scaffold_364	2	
SA_10026	scaffold_364	2	Arginine/serine-rich splicing factor, putative
SA_10026	scaffold_364	5	Arginine/serine-rich splicing factor, putative
SA_10028	scaffold_364	1	At1g23890/T23E23_13
SA_10038	scaffold_364	31	Increased size exclusion limit 2
SA_10046	scaffold_364	1	Pentatricopeptide repeat-containing protein At1g59720, mitochondrial
SA_10046	scaffold_364	2	Pentatricopeptide repeat-containing protein At1g59720, mitochondrial
SA_10046	scaffold_364	9	Pentatricopeptide repeat-containing protein At1g59720, mitochondrial
SA_10093	scaffold_364	2	Pentatricopeptide repeat-containing protein, putative
SA_10110	scaffold_364	2	Ubiquitin-protein ligase, putative
SA_10158	scaffold_364	5	RNA and export factor binding protein, putative
SA_10158	scaffold_364	6	RNA and export factor binding protein, putative

SA_10158	scaffold_364	8	RNA and export factor binding protein, putative
SA_10183	scaffold_364	9	Alpha-form rubisco activase
SA_10191	scaffold_364	5	Serine/threonine-protein phosphatase
SA_10191	scaffold_364	6	Serine/threonine-protein phosphatase
SA_10192	scaffold_370	19	Predicted protein
SA_10228	scaffold_370	3	Actin
SA_10231	scaffold_370	1	Heat shock 70kDa protein 1/8 (Fragment)
SA_10297	scaffold_370	19	DNA-directed RNA polymerase
SA_10431	scaffold_370	15	Predicted protein
SA_10476	scaffold_370	3	Glucose-6-phosphate 1-dehydrogenase
SA_10477	scaffold_370	1	Transcription factor, putative
SA_10493	scaffold_370	2	Ubiquinol-cytochrome c reductase iron-sulfur subunit
SA_10537	scaffold_379	2	Predicted protein
SA_10540	scaffold_379	4	NAC domain containing protein 57
SA_10574	scaffold_379	3	Phosphoglycerate dehydrogenase
SA_10596	scaffold_379	2	Diphthamide biosynthesis protein
SA_10597	scaffold_379	2	COR413-like protein
SA_10605	scaffold_379	12	Hydroxymethylbutenyl diphosphate reductase
SA_10605	scaffold_379	13	Hydroxymethylbutenyl diphosphate reductase
SA_10614	scaffold_379	3	Small nuclear ribonucleoprotein-like protein
SA_10631	scaffold_379	1	Chaperone protein dnaJ, putative
SA_10679	scaffold_379	10	Predicted protein
SA_10692	scaffold_379	9	Chlorophyll synthase, putative
SA_10722	scaffold_379	1	Catalytic, putative
SA_10773	scaffold_379	2	F-box/LRR-repeat protein, putative
SA_10798	scaffold_379	5	Translation initiation factor
SA_10822	scaffold_379	11	Beta-glucosidase, putative
SA_10871	scaffold_388	1	
SA_10915	scaffold_388	12	Predicted protein
SA_10915	scaffold_388	7	Predicted protein
SA_10955	scaffold_388	5	Serine/threonine protein phosphatase 2a regulatory subunit A, putative
SA_11021	scaffold_388	6	E2F, putative
SA_11058	scaffold_388	1	Nucleolar protein nop56, putative
SA_11060	scaffold_388	1	Ubiquitin-like modifier-activating enzyme
SA_11140	scaffold_394	10	Putative uncharacterized protein
SA_11154	scaffold_394	3	Bel1 homeotic protein, putative
SA_11170	scaffold_394	1	30S ribosomal protein S31, chloroplastic
SA_11174	scaffold_394	1	Uncharacterized protein
SA_11174	scaffold_394	5	Uncharacterized protein
SA_11196	scaffold_394	3	Uncharacterized protein
SA_11230	scaffold_394	32	Putative uncharacterized protein
SA_11253	scaffold_394	7	Putative uncharacterized protein

SA_11337	scaffold_394	7	Pentatricopeptide repeat-containing protein, putative
SA_11348	scaffold_394	11	Defective in cullin neddylation protein, putative
SA_11391	scaffold_394	4	Catalytic, putative
SA_11439	scaffold_394	6	Phosphatidate cytidylyltransferase
SA_11461	scaffold_394	5	Putative uncharacterized protein
SA_11470	scaffold_394	13	Putative uncharacterized protein
SA_11482	scaffold_394	2	
SA_11482	scaffold_394	3	
SA_11593	scaffold_396	5	Putative uncharacterized protein
SA_11657	scaffold_396	1	tRNA (Guanine-n(7)-)-methyltransferase, putative
SA_11666	scaffold_396	3	
SA_11680	scaffold_396	5	Triacylglycerol lipase, putative
SA_11707	scaffold_396	4	Protein SIS1, putative
SA_11710	scaffold_396	1	Protein phosphatase 2c, putative
SA_11724	scaffold_396	1	Strubbelig receptor family 3
SA_11759	scaffold_396	2	Uncharacterized protein
SA_11790	scaffold_396	6	Root phototropism protein, putative
SA_11817	scaffold_396	2	XPA-binding protein, putative
SA_11819	scaffold_396	19	Endoplasmic reticulum [ER]-type calcium ATPase
SA_11832	scaffold_396	1	TBP-associated factor 4
SA_11841	scaffold_396	10	Kinesin heavy chain, putative
SA_11855	scaffold_396	1	Protein translocase, putative
SA_11886	scaffold_396	12	Cop9 complex subunit, putative
SA_11911	scaffold_396	3	JHL18I08.15 protein
SA_11936	scaffold_396	2	JHL20J20.12 protein
SA_11941	scaffold_396	2	LYR family of Fe/S cluster biogenesis protein
SA_11962	scaffold_396	12	Integral membrane single C2 domain protein
SA_11975	scaffold_396	13	Pentatricopeptide repeat-containing protein, putative
SA_12094	scaffold_400	8	Protein kinase Ck2 regulatory subunit 2
SA_12142	scaffold_400	4	Ubiquitin-conjugating enzyme m, putative
SA_12150	scaffold_400	5	Myo inositol monophosphatase, putative
SA_12154	scaffold_400	3	Histone acetyltransferase complex component
SA_12163	scaffold_400	7	Peptidyl-prolyl cis-trans isomerase FKBP42
SA_12176	scaffold_400	1	Importin-7, putative
SA_12176	scaffold_400	2	Importin-7, putative
SA_12178	scaffold_400	15	Receptor protein kinase, putative
SA_12181	scaffold_400	1	Putative uncharacterized protein
SA_12191	scaffold_400	17	WD-repeat protein, putative
SA_12191	scaffold_400	20	WD-repeat protein, putative
SA_12200	scaffold_400	1	Uncharacterized protein
SA_12222	scaffold_400	7	Molybdopterin cofactor synthesis protein A, putative
SA_12226	scaffold_400	7	Purple acid phosphatase
SA_12239	scaffold_400	3	

SA_12296	scaffold_400	4	Putative uncharacterized protein
SA_12345	scaffold_400	18	Vacuolar proton atpase, putative
SA_12356	scaffold_400	4	
SA_12371	scaffold_400	4	Protein Z, putative
SA_12384	scaffold_400	6	Serine/threonine-protein kinase PBS1, putative
SA_12408	scaffold_400	3	Hspc200, putative
SA_12450	scaffold_400	1	Phosphoglycerate mutase, putative
SA_12497	scaffold_403	9	Superoxide dismutase [Mn], mitochondrial
SA_12515	scaffold_407	12	Protein GPR89A, putative
SA_12518	scaffold_407	9	GTP pyrophosphokinase
SA_12523	scaffold_407	3	Putative uncharacterized protein
SA_12534	scaffold_407	14	Catalytic, putative
SA_12546	scaffold_407	11	Pentatricopeptide repeat-containing protein
SA_12546	scaffold_407	12	Pentatricopeptide repeat-containing protein
SA_12563	scaffold_407	2	Predicted protein
SA_12584	scaffold_407	1	60S ribosomal protein L36
SA_12591	scaffold_407	2	Ribosomal protein S9, putative
SA_12596	scaffold_407	2	Beta-1,3-galactosyltransferase sqv-2, putative
SA_12598	scaffold_407	1	Ubiquitin-conjugating enzyme E2
SA_12598	scaffold_407	3	Ubiquitin-conjugating enzyme E2
SA_12609	scaffold_407	1	Calcineurin B-like protein 04
SA_12624	scaffold_407	1	60S acidic ribosomal protein P1, putative
SA_12677	scaffold_407	4	Oligopeptide transporter, putative
SA_12688	scaffold_407	3	Microfibrillar-associated protein
SA_12712	scaffold_407	1	Uncharacterized protein
SA_12750	scaffold_407	6	Transcription factor RF2a, putative
SA_12768	scaffold_407	7	Protein kinase family protein
SA_12768	scaffold_407	8	Protein kinase family protein
SA_12784	scaffold_407	4	ARF domain class transcription factor
SA_12800	scaffold_407	4	40S ribosomal protein S12
SA_12800	scaffold_407	5	40S ribosomal protein S12
SA_12875	scaffold_407	1	Ubiquitin-protein ligase, putative
SA_12891	scaffold_407	3	Predicted protein
SA_12915	scaffold_407	2	Predicted protein
SA_12928	scaffold_407	9	Calmodulin binding protein, putative
SA_12961	scaffold_407	6	Putative uncharacterized protein
SA_12961	scaffold_407	7	Putative uncharacterized protein
SA_12966	scaffold_407	1	Polyadenylate-binding protein, putative
SA_13009	scaffold_407	2	Putative uncharacterized protein
SA_13021	scaffold_407	1	At4g24330
SA_13098	scaffold_655	7	KH domain-containing protein
SA_13108	scaffold_655	5	Organic anion transporter, putative
SA_13113	scaffold_655	8	Electron transporter, putative

SA_13113	scaffold_655	9	Electron transporter, putative
SA_13135	scaffold_655	7	Calcium ion binding protein, putative
SA_13136	scaffold_655	5	Protein kinase
SA_13157	scaffold_655	1	
SA_13210	scaffold_686	1	RNA-binding protein, putative
SA_13212	scaffold_686	2	RNA-binding protein, putative
SA_13226	scaffold_686	1	AGAP006961-PA
SA_13259	scaffold_686	5	Putative uncharacterized protein
SA_13261	scaffold_686	8	Protein phosphatase 2c, putative
SA_13292	scaffold_705	3	Uncharacterized protein
SA_13313	scaffold_705	1	Predicted protein
SA_13392	scaffold_705	5	Leucine-rich repeat receptor protein kinase EXS, putative
SA_13392	scaffold_705	6	Leucine-rich repeat receptor protein kinase EXS, putative
SA_13403	scaffold_705	1	Putative uncharacterized protein
SA_13404	scaffold_705	6	Uncharacterized protein
SA_13410	scaffold_705	1	Putative uncharacterized protein
SA_13527	scaffold_705	1	CDPK-related protein kinase
SA_13531	scaffold_705	18	Binding protein
SA_13614	scaffold_705	12	Bel1 homeotic protein, putative
SA_13615	scaffold_705	15	Arsenical pump-driving atpase, putative
SA_13624	scaffold_705	1	
SA_13659	scaffold_705	10	tRNA delta(2)-isopentenylpyrophosphate transferase, putative
SA_13671	scaffold_705	4	Catalytic, putative
SA_13703	scaffold_705	4	Molybdopterin biosynthesis protein, putative
SA_13715	scaffold_705	3	ABSCISIC ACID-INSENSITIVE 5-like protein
SA_13719	scaffold_705	1	CBL-interacting serine/threonine-protein kinase, putative
SA_13750	scaffold_705	5	Poly(P)/ATP NAD kinase, putative
SA_13788	scaffold_705	1	F-box/kelch-repeat protein
SA_13832	scaffold_705	18	Calnexin, putative
SA_13926	scaffold_705	3	D-alanyl-D-alanine endopeptidase, putative
SA_13955	scaffold_705	2	Derlin-2, putative
SA_13996	scaffold_705	1	Putative uncharacterized protein
SA_14052	scaffold_705	1	Predicted protein
SA_14060	scaffold_705	1	Eukaryotic translation initiation factor 3 subunit, putative
SA_14095	scaffold_795	2	SUN domain-containing protein
SA_14116	scaffold_795	12	Putative uncharacterized protein
SA_14130	scaffold_795	3	R3h domain containing protein, putative
SA_14186	scaffold_795	2	Heat shock 70kDa protein 1/8
SA_14211	scaffold_795	2	
SA_14233	scaffold_795	2	Predicted protein

SA_14233	scaffold_795	4	Predicted protein
SA_14324	scaffold_795	1	Ubiquitin-protein ligase, putative
SA_14324	scaffold_795	11	Ubiquitin-protein ligase, putative
SA_14403	scaffold_795	4	
SA_14424	scaffold_795	8	Protein phosphatase 2a, regulatory subunit, putative
SA_14527	scaffold_795	6	mRNA, clone: RTFL01-46-J02
SA_14543	scaffold_795	6	Putative uncharacterized protein
SA_14575	scaffold_795	2	Os05g0372100 protein
SA_14578	scaffold_795	9	Uncharacterized protein
SA_14639	scaffold_795	3	Putative uncharacterized protein
SA_14659	scaffold_795	1	Protein C20orf4, putative
SA_14668	scaffold_795	1	ATA15 protein
SA_14682	scaffold_795	2	Protease C56, putative
SA_14703	scaffold_795	2	50S ribosomal protein L28, chloroplast, putative
SA_14806	scaffold_891	3	Putative uncharacterized protein
SA_14841	scaffold_891	6	Vacuolar ATP synthase subunit E, putative
SA_14845	scaffold_891	3	24 kDa seed maturation protein
SA_14860	scaffold_891	4	Ubiquitin-conjugating enzyme h, putative
SA_14884	scaffold_891	6	F-box/kelch-repeat protein
SA_14897	scaffold_891	21	EXECUTER1 protein, chloroplast, putative
SA_14963	scaffold_891	6	Predicted protein
SA_14971	scaffold_891	1	Calcium/calmodulin-dependent protein kinase CaMK3
SA_14971	scaffold_891	2	Calcium/calmodulin-dependent protein kinase CaMK3
SA_14989	scaffold_891	2	1-acyl-sn-glycerol-3-phosphate acyltransferase
SA_14997	scaffold_893	1	Putative uncharacterized protein
SA_15015	scaffold_893	3	Homeobox protein knotted-1-like 3
SA_15017	scaffold_893	1	Serine-rich protein
SA_15034	scaffold_893	4	Putative uncharacterized protein
SA_15070	scaffold_893	11	Eukaryotic translation initiation factor 3 subunit L
SA_15165	scaffold_893	19	ABC1 family protein
SA_15174	scaffold_893	11	DNA binding protein, putative
SA_15174	scaffold_893	4	DNA binding protein, putative
SA_15183	scaffold_893	14	Eukaryotic translation initiation factor 3 subunit, putative
SA_15185	scaffold_893	2	Predicted protein
SA_15205	scaffold_893	4	Putative uncharacterized protein
SA_15255	scaffold_893	13	Putative uncharacterized protein
SA_15261	scaffold_893	4	Fad NAD binding oxidoreductases, putative
SA_15277	scaffold_893	1	ESC, putative
SA_15280	scaffold_893	1	
SA_15280	scaffold_893	2	
SA_15355	scaffold_893	7	Exocyst complex component EXO70
SA_15378	scaffold_893	1	E3 ubiquitin-protein ligase RING1

SA_15390	scaffold_893	1	DNA binding protein, putative
SA_15400	scaffold_893	4	3-ketoacyl-ACP synthase
SA_15402	scaffold_893	5	Groes chaperonin, putative
SA_15404	scaffold_893	1	Putative uncharacterized protein
SA_15410	scaffold_893	6	Putative uncharacterized protein
SA_15413	scaffold_893	12	Cation:cation antiporter, putative
SA_15413	scaffold_893	13	Cation:cation antiporter, putative
SA_15438	scaffold_893	6	DECOY, putative
SA_15450	scaffold_893	18	Putative uncharacterized protein
SA_15486	scaffold_893	14	Leucine-rich repeat protein kinase
SA_15486	scaffold_893	16	Leucine-rich repeat protein kinase
SA_15611	scaffold_893	1	Transcription factor, putative
SA_15611	scaffold_893	12	Transcription factor, putative
SA_15636	scaffold_893	3	Pyruvate kinase
SA_15639	scaffold_893	16	Oxidoreductase, 2OG-Fe(II) oxygenase family protein
SA_15652	scaffold_893	4	Homeodomain leucine zipper protein
SA_15663	scaffold_893	5	Cinnamoyl-CoA reductase
SA_15699	scaffold_893	5	Predicted protein
SA_15714	scaffold_893	10	Metalloendopeptidase, putative
SA_15715	scaffold_893	6	Mitochondrial import receptor subunit TOM20
SA_15715	scaffold_893	7	Mitochondrial import receptor subunit TOM20
SA_15730	scaffold_893	4	Putative uncharacterized protein
SA_15735	scaffold_893	10	Putative uncharacterized protein
SA_15735	scaffold_893	6	Putative uncharacterized protein
SA_15749	scaffold_893	3	40S ribosomal protein S27
SA_15755	scaffold_893	1	Predicted protein
SA_15755	scaffold_893	2	Predicted protein
SA_15826	scaffold_897	4	DNA cross-link repair protein pso2/snm1, putative
SA_15843	scaffold_897	8	Putative uncharacterized protein
SA_15843	scaffold_897	9	Putative uncharacterized protein
SA_15887	scaffold_897	5	Predicted protein
SA_15905	scaffold_897	1	Glycosyltransferase QUASIMODO1, putative
SA_15910	scaffold_897	15	Big map kinase/bmk, putative
SA_15910	scaffold_897	3	Big map kinase/bmk, putative
SA_15910	scaffold_897	5	Big map kinase/bmk, putative
SA_15955	scaffold_897	7	Cap-binding protein-like protein
SA_15955	scaffold_897	8	Cap-binding protein-like protein
SA_15997	scaffold_897	5	Vesicle-associated membrane family protein
SA_16009	scaffold_897	6	Uncharacterized protein
SA_16049	scaffold_897	7	Probable methyltransferase PMT13
SA_16059	scaffold_897	8	DNA binding protein, putative
SA_16080	scaffold_897	20	Nucleic acid binding protein, putative
SA_16255	scaffold_899	2	Putative uncharacterized protein

SA_16340	scaffold_899	30	Beta-glucosidase, putative
SA_16348	scaffold_899	2	ETHYLENE-INSENSITIVE3 protein, putative
SA_16367	scaffold_899	13	Ubiquitin carboxyl-terminal hydrolase
SA_16423	scaffold_899	4	Putative uncharacterized protein
SA_16516	scaffold_899	2	Putative uncharacterized protein
SA_16545	scaffold_899	11	Putative uncharacterized protein
SA_16568	scaffold_899	8	ROP1.1 (Fragment)
SA_16575	scaffold_899	6	RING-H2 finger protein ATL4M, putative
SA_16595	scaffold_899	7	Srpk, putative
SA_16604	scaffold_899	7	Putative uncharacterized protein
SA_16608	scaffold_899	2	Autoinhibited H+ ATPase
SA_16613	scaffold_899	2	AP2 domain class transcription factor
SA_16621	scaffold_899	5	25.3 kDa vesicle transport protein
SA_16628	scaffold_899	2	Pentatricopeptide repeat-containing protein, putative
SA_16628	scaffold_899	5	Pentatricopeptide repeat-containing protein, putative
SA_16629	scaffold_899	1	Protein binding protein, putative
SA_16629	scaffold_899	2	Protein binding protein, putative
SA_16699	scaffold_899	2	Uncharacterized protein
SA_16721	scaffold_899	3	Vacuolar protein sorting protein, putative
SA_16731	scaffold_899	10	Putative uncharacterized protein
SA_16731	scaffold_899	8	Putative uncharacterized protein
SA_16732	scaffold_899	1	Putative uncharacterized protein
SA_16753	scaffold_899	6	Kinase-like protein (Fragment)
SA_16766	scaffold_899	2	
SA_16806	scaffold_899	9	Mitochondrial carrier protein, putative
SA_16854	scaffold_899	16	MAF1-like protein
SA_16895	scaffold_899	8	Phosphopantothenate-cysteine ligase
SA_16921	scaffold_899	8	Putative uncharacterized protein
SA_16960	scaffold_899	2	Uncharacterized protein
SA_17059	scaffold_900	11	Zinc binding dehydrogenase, putative
SA_17090	scaffold_900	1	Putative uncharacterized protein
SA_17162	scaffold_913	4	Uncharacterized protein
SA_17183	scaffold_913	6	Putative uncharacterized protein
SA_17197	scaffold_913	4	E3 ubiquitin-protein ligase CHFR
SA_17340	scaffold_913	1	Putative uncharacterized protein
SA_17396	scaffold_913	1	Cytochrome P450
SA_17396	scaffold_913	7	Cytochrome P450
SA_17420	scaffold_916	3	Hsp70-interacting protein 1
SA_17483	scaffold_916	5	Putative uncharacterized protein
SA_17554	scaffold_916	7	Tetratricopeptide repeat (TPR)-containing protein
SA_17570	scaffold_916	2	Putative uncharacterized protein
SA_17570	scaffold_916	3	Putative uncharacterized protein
SA_17613	scaffold_916	10	Predicted protein (Fragment)

SA_17613	scaffold_916	7	Predicted protein (Fragment)
SA_17636	scaffold_916	6	Xyloglucan galactosyltransferase
SA_17671	scaffold_916	10	Peptide-N4-(N-acetyl-beta-glucosaminyl)asparagine amidase A
SA_17671	scaffold_916	9	Peptide-N4-(N-acetyl-beta-glucosaminyl)asparagine amidase A
SA_17680	scaffold_916	11	Uncharacterized protein
SA_17688	scaffold_916	3	Protein phosphatase 2c, putative
SA_17695	scaffold_916	2	Uncharacterized protein
SA_17713	scaffold_916	26	Myosin vIII, putative
SA_17782	scaffold_916	12	Protein transport protein Sec24A, putative
SA_17782	scaffold_916	14	Protein transport protein Sec24A, putative
SA_17784	scaffold_916	1	Serine acetyltransferase 3, mitochondrial, putative
SA_17823	scaffold_916	3	Putative uncharacterized protein
SA_17855	scaffold_916	2	
SA_17880	scaffold_916	9	AT4g08960/T3H13_2
SA_17896	scaffold_916	3	Transcription factor, putative
SA_17896	scaffold_916	5	Transcription factor, putative
SA_17943	scaffold_916	5	F10A16.24 protein
SA_17984	scaffold_916	1	26S protease regulatory subunit 6A-like protein
SA_17984	scaffold_916	12	26S protease regulatory subunit 6A-like protein
SA_17984	scaffold_916	13	26S protease regulatory subunit 6A-like protein
SA_17984	scaffold_916	3	26S protease regulatory subunit 6A-like protein
SA_17996	scaffold_916	1	UDP-N-acetylglucosamine transferase subunit alg13, putative
SA_17998	scaffold_916	2	BHLH domain class transcription factor
SA_18019	scaffold_916	5	V-type proton ATPase subunit F
SA_18022	scaffold_916	5	Glutamyl-tRNA synthetase
SA_18036	scaffold_916	1	ERD15
SA_18048	scaffold_916	7	AT3G10250 protein
SA_18127	scaffold_916	5	Exoenzymes regulatory protein aepA, putative
SA_18134	scaffold_916	14	U3 small nucleolar ribonucleoprotein protein imp4, putative
SA_18143	scaffold_916	9	Predicted protein
SA_18147	scaffold_916	3	Putative uncharacterized protein
SA_18161	scaffold_916	1	tRNA/rRNA methyltransferase, putative
SA_18231	scaffold_917	4	Eyes absent-like protein
SA_18248	scaffold_917	20	Predicted protein
SA_18257	scaffold_917	4	RNA binding protein, putative
SA_18257	scaffold_917	9	RNA binding protein, putative
SA_18295	scaffold_917	4	Leucine rich repeat receptor kinase, putative
SA_18358	scaffold_917	10	Serine/threonine-protein kinase PEPKR2
SA_18381	scaffold_917	1	Adenylate kinase
SA_18428	scaffold_917	1	DNA double-strand break repair rad50 ATPase, putative
SA_18481	scaffold_917	1	Putative uncharacterized protein

SA_18483	scaffold_917	11	Putative uncharacterized protein
SA_18512	scaffold_917	7	Putative uncharacterized protein
SA_18546	scaffold_917	1	Pentatricopeptide repeat-containing protein, putative
SA_18560	scaffold_917	14	GIGANTEA
SA_18562	scaffold_917	7	Pentatricopeptide repeat-containing protein, putative
SA_18567	scaffold_917	4	Putative uncharacterized protein
SA_18583	scaffold_917	11	Predicted protein
SA_18583	scaffold_917	7	Predicted protein
SA_18583	scaffold_917	8	Predicted protein
SA_18596	scaffold_917	9	Protein transporter, putative
SA_18599	scaffold_917	39	F-box and wd40 domain protein, putative
SA_18615	scaffold_917	6	Neutral/alkaline invertase
SA_18654	scaffold_917	2	Pentatricopeptide repeat-containing protein
SA_18659	scaffold_917	9	Putative uncharacterized protein
SA_18701	scaffold_917	1	Pre-mRNA-splicing factor cwc23, putative
SA_18708	scaffold_917	4	Peptidyl-prolyl cis-trans isomerase
SA_18753	scaffold_917	1	Glycine-rich RNA-binding protein GRP1A, putative, expressed
SA_18762	scaffold_917	6	Uncharacterized protein
SA_18770	scaffold_917	4	COR413-PM2, putative
SA_18783	scaffold_917	12	CPK related kinase 5
SA_18783	scaffold_917	8	CPK related kinase 5
SA_18821	scaffold_917	9	Putative uncharacterized protein
SA_18852	scaffold_917	3	Magnesium-dependent phosphatase, putative
SA_18867	scaffold_917	6	2-nitropropane dioxygenase, putative
SA_18874	scaffold_917	18	Endoplasmic reticulum-Golgi intermediate compartment protein, putative
SA_18880	scaffold_917	8	Superoxide dismutase copper chaperone, putative
SA_18880	scaffold_917	9	Superoxide dismutase copper chaperone, putative
SA_18924	scaffold_917	14	Respiratory burst oxidase, putative
SA_18928	scaffold_917	1	Transcription factor, putative
SA_18928	scaffold_917	2	Transcription factor, putative
SA_18964	scaffold_917	14	Putative uncharacterized protein
SA_19000	scaffold_917	10	Pectinacetylesterase (Precursor)
SA_19000	scaffold_917	11	Pectinacetylesterase (Precursor)
SA_19000	scaffold_917	9	Pectinacetylesterase (Precursor)
SA_19037	scaffold_917	4	Dihydrodipicolinate synthase, putative
SA_19082	scaffold_918	13	Translation initiation factor eIF-2B subunit gamma
SA_19094	scaffold_918	14	La-related protein
SA_19129	scaffold_918	1	Ferredoxin-3, chloroplast, putative
SA_19146	scaffold_918	6	Expressed protein
SA_19149	scaffold_918	10	Aspartyl protease family protein
SA_19167	scaffold_918	1	Lactoylglutathione lyase
SA_19167	scaffold_918	2	Lactoylglutathione lyase

SA_19188	scaffold_918	6	Heat shock protein 70 (HSP70)-interacting protein, putative
SA_19219	scaffold_918	11	At1g68370
SA_19244	scaffold_918	3	Predicted protein (Fragment)
SA_19246	scaffold_918	15	Clathrin assembly protein, putative
SA_19270	scaffold_918	2	Serine/threonine-protein kinase
SA_19294	scaffold_918	5	Two-component system sensor histidine kinase/response regulator, putative
SA_19309	scaffold_918	9	Translation initiation factor IF-3
SA_19342	scaffold_918	1	Serine/threonine protein kinase, putative
SA_19572	scaffold_918	21	Aspartic proteinase nepenthesin-2, putative
SA_19635	scaffold_918	1	Predicted protein
SA_19706	scaffold_918	5	Probable mannitol dehydrogenase
SA_19710	scaffold_918	6	Heat shock 70 kDa protein, mitochondrial
SA_19788	scaffold_918	1	
SA_19802	scaffold_918	1	Mitochondrial carrier protein, putative
SA_19821	scaffold_918	3	Protein trichome berefringence-like 7
SA_19855	scaffold_918	12	Putative uncharacterized protein
SA_19874	scaffold_918	31	Transcription cofactor, putative
SA_19886	scaffold_918	10	Mannosyl-oligosaccharide 1,2-alpha-mannosidase (Fragment)
SA_19886	scaffold_918	24	Mannosyl-oligosaccharide 1,2-alpha-mannosidase (Fragment)
SA_19921	scaffold_918	3	
SA_19956	scaffold_918	2	NMDA receptor-regulated protein, putative
SA_19957	scaffold_918	7	ATP binding protein, putative
SA_20000	scaffold_918	27	Putative uncharacterized protein
SA_20033	scaffold_918	1	SEC14 cytosolic factor, putative
SA_20215	scaffold_918	1	
SA_20225	scaffold_919	4	Actin-related protein 2/3 complex subunit 5
SA_20286	scaffold_919	1	
SA_20372	scaffold_921	1	Mads box protein, putative
SA_20372	scaffold_921	4	Mads box protein, putative
SA_20388	scaffold_921	17	Dihydrolipoamide dehydrogenase, putative
SA_20412	scaffold_921	9	Putative uncharacterized protein
SA_20420	scaffold_921	6	Putative uncharacterized protein
SA_20437	scaffold_922	1	AT4g28240/F26K10_120
SA_20459	scaffold_922	1	Hydrolase, hydrolyzing O-glycosyl compounds, putative
SA_20465	scaffold_922	12	Protein binding protein, putative
SA_20490	scaffold_922	4	ATMBF1B/MBF1B
SA_20552	scaffold_922	5	Putative uncharacterized protein
SA_20568	scaffold_922	13	TGA transcription factor
SA_20591	scaffold_922	7	Uncharacterized protein
SA_20636	scaffold_922	17	O-linked n-acetylglucosamine transferase, ogt, putative
SA_20692	scaffold_922	7	Fuct c3 protein

SA_20714	scaffold_922	15	Auxin response factor, putative
SA_20714	scaffold_922	19	Auxin response factor, putative
SA_20719	scaffold_922	1	Putative uncharacterized protein
SA_20726	scaffold_922	7	Nucleic acid binding protein, putative
SA_20741	scaffold_922	3	Magnesium transporter MRS2
SA_20770	scaffold_922	15	Potassium transporter, putative
SA_20807	scaffold_922	19	Cactin (ISS)
SA_20809	scaffold_922	1	Aquaporin
SA_20818	scaffold_922	2	Histidyl-tRNA synthetase, putative
SA_20869	scaffold_922	5	40S ribosomal protein S3a-like protein
SA_20895	scaffold_922	1	Predicted protein
SA_20910	scaffold_922	10	Potassium transporter, putative
SA_20933	scaffold_922	5	Putative uncharacterized protein
SA_20935	scaffold_922	11	Receptor protein kinase, putative
SA_20959	scaffold_922	1	Fasciclin-like domain-containing protein
SA_21095	scaffold_922	6	NAC domain protein, IPR003441 (Fragment)
SA_21110	scaffold_922	1	Putative uncharacterized protein
SA_21158	scaffold_922	1	Glucose regulated repressor protein, putative
SA_21193	scaffold_922	1	Dynamin
SA_21210	scaffold_922	1	Lupus la ribonucleoprotein, putative
SA_21224	scaffold_922	9	Eukaryotic translation initiation factor 3 subunit, putative
SA_21269	scaffold_922	1	
SA_21357	scaffold_923	5	Dihydrolipoamide succinyltransferase component of 2-oxoglutarate dehydrogenase, putative
SA_21363	scaffold_923	3	VTC2-like protein
SA_21372	scaffold_923	1	DNA topoisomerase type I, putative
SA_21396	scaffold_923	15	Proliferating-cell nucleolar antigen p120, putative
SA_21403	scaffold_923	1	AGAP006959-PA
SA_21403	scaffold_923	2	AGAP006959-PA
SA_21404	scaffold_923	2	UV-induced protein uvi31, putative
SA_21449	scaffold_923	9	PsbP domain-containing protein 3, chloroplastic
SA_21457	scaffold_923	2	Ribophorin, putative
SA_21458	scaffold_923	3	Dolichyl-diphosphooligosaccharide-protein glycosyltransferase subunit
SA_21585	scaffold_923	16	Type-1 phosphatidic acid phosphohydrolase 2
SA_21590	scaffold_923	11	Acyl-peptide hydrolase-like protein
SA_21607	scaffold_923	9	Predicted protein
SA_21621	scaffold_923	3	
SA_21621	scaffold_923	4	
SA_21624	scaffold_923	9	ATP binding protein, putative
SA_21646	scaffold_923	9	Salt-tolerance protein, putative
SA_21670	scaffold_923	7	Heat Shock Protein 90, endoplasmic reticulum
SA_21700	scaffold_923	1	Putative uncharacterized protein

SA_21759	scaffold_923	5	Predicted protein
SA_21789	scaffold_923	1	DNA binding protein, putative
SA_21880	scaffold_923	4	Probably inactive leucine-rich repeat receptor-like protein kinase At3g28040
SA_21881	scaffold_923	1	
SA_21906	scaffold_923	15	Uncharacterized protein
SA_21907	scaffold_923	1	Putative uncharacterized protein
SA_21928	scaffold_923	10	TBC1 domain family member 22A
SA_21943	scaffold_923	3	Protein kinase
SA_21966	scaffold_923	4	Nucleic acid binding protein, putative
SA_22009	scaffold_923	20	Putative uncharacterized protein
SA_22014	scaffold_923	6	Korrigan
SA_22079	scaffold_923	5	Heat shock protein 70 (HSP70)-interacting protein, putative
SA_22079	scaffold_923	6	Heat shock protein 70 (HSP70)-interacting protein, putative
SA_22121	scaffold_923	2	Predicted protein
SA_22193	scaffold_925	4	
SA_22213	scaffold_925	5	Signalosome subunit, putative
SA_22257	scaffold_925	11	SET domain-containing protein (Fragment)
SA_22264	scaffold_925	1	Predicted protein
SA_22298	scaffold_925	2	Taxane 13-alpha-hydroxylase
SA_22317	scaffold_925	4	Nitrate transporter, putative
SA_22389	scaffold_925	5	DNA mismatch repair protein MSH2, putative
SA_22431	scaffold_925	2	Phosphoethanolamine n-methyltransferase, putative
SA_22437	scaffold_925	4	Putative uncharacterized protein
SA_22601	scaffold_925	5	Uncharacterized protein
SA_22618	scaffold_925	5	Serine/threonine-protein phosphatase
SA_22689	scaffold_925	1	Putative uncharacterized protein
SA_22689	scaffold_925	4	Putative uncharacterized protein
SA_22706	scaffold_925	2	JHL07K02.9 protein
SA_22721	scaffold_927	7	Os07g0295200 protein
SA_22831	scaffold_927	23	Heat shock protein binding protein, putative
SA_22831	scaffold_927	25	Heat shock protein binding protein, putative
SA_22837	scaffold_927	11	Formin-like protein 20
SA_22837	scaffold_927	12	Formin-like protein 20
SA_22866	scaffold_927	3	Putative uncharacterized protein
SA_22872	scaffold_927	5	JHL10I11.3 protein
SA_22934	scaffold_927	8	Down syndrome critical region protein-like protein
SA_22952	scaffold_927	1	Predicted protein
SA_22979	scaffold_927	4	GEM-like protein
SA_22988	scaffold_927	9	XPA-binding protein, putative
SA_22989	scaffold_927	7	RNA binding protein, putative
SA_23006	scaffold_927	1	GPI-anchor transamidase, putative

SA_23027	scaffold_927	4	Protein ABC1, mitochondrial, putative
SA_23030	scaffold_927	6	Fasciclin-like arabinogalactan protein 19
SA_23061	scaffold_927	3	At1g57540
SA_23064	scaffold_927	1	
SA_23086	scaffold_927	3	Translation initiation factor eif-2b beta subunit, putative
SA_23105	scaffold_927	1	Mevalonate kinase
SA_23160	scaffold_927	5	Early-responsive to dehydration
SA_23169	scaffold_927	20	Class III HD-Zip protein 4
SA_23242	scaffold_927	1	Copine, putative
SA_23244	scaffold_927	39	Sugar transporter, putative
SA_23294	scaffold_927	10	Pentatricopeptide repeat-containing protein, putative
SA_23305	scaffold_927	4	Putative uncharacterized protein
SA_23305	scaffold_927	6	Putative uncharacterized protein
SA_23321	scaffold_927	2	Serine/threonine-protein kinase PBS1, putative
SA_23326	scaffold_927	1	WD-repeat protein, putative
SA_23361	scaffold_927	1	Pentatricopeptide repeat-containing protein, putative
SA_23372	scaffold_927	8	Ribonuclease, putative
SA_23385	scaffold_927	1	RNA binding protein, putative
SA_23489	scaffold_929	8	SINA1
SA_23491	scaffold_929	3	Carboxylesterase np, putative
SA_23591	scaffold_929	2	Transcription factor, putative
SA_23619	scaffold_929	1	Uncharacterized protein
SA_23640	scaffold_929	5	LRR receptor-like protein kinase
SA_23661	scaffold_929	1	Protein YME1, putative
SA_23662	scaffold_929	3	UDP-sulfoquinovose synthase, putative
SA_23679	scaffold_929	2	Putative uncharacterized protein
SA_23772	scaffold_929	3	2-hydroxyacid dehydrogenase, putative
SA_23772	scaffold_929	5	2-hydroxyacid dehydrogenase, putative
SA_23841	scaffold_929	1	Uncharacterized protein
SA_23851	scaffold_929	1	Putative uncharacterized protein
SA_23884	scaffold_929	6	
SA_23962	scaffold_929	4	Putative uncharacterized protein
SA_23999	scaffold_929	7	Uncharacterized protein
SA_24007	scaffold_929	2	Predicted protein
SA_24007	scaffold_929	7	Predicted protein
SA_24020	scaffold_929	1	S-adenosyl-L-methionine:delta24-sterol-C-methyltransferase
SA_24020	scaffold_929	2	S-adenosyl-L-methionine:delta24-sterol-C-methyltransferase
SA_24020	scaffold_929	3	S-adenosyl-L-methionine:delta24-sterol-C-methyltransferase
SA_24025	scaffold_929	10	Cellulose synthase catalytic subunit
SA_24036	scaffold_929	19	Citrate synthase
SA_24036	scaffold_929	20	Citrate synthase

SA_24119	scaffold_929	1	N-rich protein, putative
SA_24119	scaffold_929	2	N-rich protein, putative
SA_24119	scaffold_929	3	N-rich protein, putative
SA_24133	scaffold_929	1	Nut2, putative
SA_24154	scaffold_929	1	
SA_24175	scaffold_929	1	DNA polymerase III subunit gamma/tau
SA_24186	scaffold_929	3	Uncharacterized protein
SA_24198	scaffold_929	23	Anaphase-promoting complex subunit, putative
SA_24205	scaffold_929	1	Pentatricopeptide repeat-containing protein, putative
SA_24213	scaffold_929	2	BRASSINOSTEROID INSENSITIVE 1, putative
SA_24225	scaffold_929	1	Uncharacterized protein
SA_24239	scaffold_929	4	Predicted protein
SA_24289	scaffold_930	4	Thioredoxin-like 3-2, chloroplastic
SA_24290	scaffold_930	4	Amino acid transporter
SA_24298	scaffold_930	18	3-phosphoinositide-dependent protein kinase-1, putative
SA_24353	scaffold_930	49	MAPKKK7
SA_24393	scaffold_930	7	Polysialic acid capsule expression protein kpsF, putative
SA_24403	scaffold_930	5	JHL20J20.13 protein
SA_24416	scaffold_930	1	Putative uncharacterized protein
SA_24448	scaffold_930	3	Tubulin-specific chaperone A (Tubulin-folding cofactor A) (Cfa), putative
SA_24461	scaffold_930	2	Pentatricopeptide repeat-containing protein, putative
SA_24461	scaffold_930	4	Pentatricopeptide repeat-containing protein, putative
SA_24516	scaffold_930	3	DNA methyltransferase 1-associated protein, putative
SA_24585	scaffold_930	1	Putative uncharacterized protein
SA_24602	scaffold_930	2	Putative uncharacterized protein
SA_24610	scaffold_930	2	Protein phosphatase 2c, putative
SA_24610	scaffold_930	9	Protein phosphatase 2c, putative
SA_24698	scaffold_930	13	Predicted protein (Fragment)
SA_24730	scaffold_930	19	Amino acid transporter, putative
SA_24730	scaffold_930	24	Amino acid transporter, putative
SA_24730	scaffold_930	25	Amino acid transporter, putative
SA_24730	scaffold_930	27	Amino acid transporter, putative
SA_24730	scaffold_930	28	Amino acid transporter, putative
SA_24730	scaffold_930	29	Amino acid transporter, putative
SA_24748	scaffold_930	5	Ubiquitin-conjugating enzyme E2-25kD, putative
SA_24763	scaffold_930	1	Receptor serine-threonine protein kinase, putative
SA_24803	scaffold_930	1	Long-chain acyl-CoA synthetase 1
SA_24808	scaffold_930	2	Transmembrane emp24 domain-containing protein 10, putative
SA_24837	scaffold_930	1	
SA_24912	scaffold_930	19	Protein binding protein, putative
SA_24929	scaffold_930	2	
SA_24930	scaffold_930	2	

SA_24930	scaffold_930	3	
SA_25003	scaffold_930	17	Putative uncharacterized protein
SA_25018	scaffold_930	1	
SA_25082	scaffold_930	1	Serine/threonine-protein phosphatase
SA_25101	scaffold_930	4	Aux/IAA protein
SA_25130	scaffold_930	4	SCARECROW
SA_25134	scaffold_930	1	
SA_25141	scaffold_930	1	Predicted protein (Fragment)
SA_25148	scaffold_930	3	Uncharacterized protein
SA_25182	scaffold_930	1	Fructokinase-like protein 1
SA_25206	scaffold_930	3	Uncharacterized protein
SA_25251	scaffold_930	1	Transcription factor, putative
SA_25280	scaffold_930	3	Os07g0405100 protein
SA_25342	scaffold_930	3	Stress enhanced protein 2
SA_25365	scaffold_930	18	Ubiquitin ligase E3 alpha, putative
SA_25365	scaffold_930	19	Ubiquitin ligase E3 alpha, putative
SA_25365	scaffold_930	2	Ubiquitin ligase E3 alpha, putative
SA_25368	scaffold_930	1	Metallothionein-like protein
SA_25369	scaffold_930	1	
SA_25374	scaffold_930	10	Putative uncharacterized protein
SA_25374	scaffold_930	18	Putative uncharacterized protein
SA_25405	scaffold_930	2	White-brown-complex ABC transporter family
SA_25418	scaffold_930	16	Dead box ATP-dependent RNA helicase, putative
SA_25425	scaffold_930	2	Nucleic acid binding protein, putative
SA_25476	scaffold_930	2	
SA_25517	scaffold_930	2	Bel1 homeotic protein, putative
SA_25530	scaffold_930	7	Uncharacterized protein
SA_25534	scaffold_930	5	
SA_25584	scaffold_934	2	Putative uncharacterized protein
SA_25588	scaffold_934	1	NADPH--cytochrome P450 reductase
SA_25612	scaffold_934	5	Predicted protein
SA_25616	scaffold_934	15	Biotin carboxylase 2, chloroplastic
SA_25625	scaffold_934	20	UDP-sugar transporter, putative
SA_25625	scaffold_934	21	UDP-sugar transporter, putative
SA_25625	scaffold_934	26	UDP-sugar transporter, putative
SA_25636	scaffold_934	3	
SA_25648	scaffold_934	7	Putative uncharacterized protein
SA_25702	scaffold_934	2	Putative uncharacterized protein
SA_25713	scaffold_934	2	C3HL domain class transcription factor
SA_25738	scaffold_934	1	Protein phosphatase 2c
SA_25790	scaffold_934	10	Putative uncharacterized protein
SA_25803	scaffold_934	1	Protein ABC1, mitochondrial, putative
SA_25899	scaffold_934	3	Eukaryotic translation initiation factor 3f, eif3f, putative

SA_25902	scaffold_934	3	Pollen-specific protein SF3, putative
SA_25926	scaffold_935	12	Putative uncharacterized protein
SA_25926	scaffold_935	4	Putative uncharacterized protein
SA_25929	scaffold_935	3	Protein phosphatase 2c, putative
SA_25933	scaffold_935	12	Endoplasmic reticulum-Golgi intermediate compartment protein, putative
SA_25933	scaffold_935	16	Endoplasmic reticulum-Golgi intermediate compartment protein, putative
SA_25948	scaffold_935	14	Map3k delta-1 protein kinase, putative
SA_25957	scaffold_935	2	AMP dependent CoA ligase, putative
SA_25964	scaffold_935	9	At4g23390
SA_25973	scaffold_935	9	Transferase, transferring glycosyl groups, putative
SA_25994	scaffold_935	7	Predicted protein
SA_26016	scaffold_935	4	Protein ABIL2, putative
SA_26042	scaffold_935	4	NAC domain protein, IPR003441 (Fragment)
SA_26050	scaffold_935	9	F-box protein SKIP31
SA_26101	scaffold_935	1	Cytohesin 1, 2, 3, putative
SA_26101	scaffold_935	3	Cytohesin 1, 2, 3, putative
SA_26130	scaffold_938	14	Os02g0537900 protein
SA_26172	scaffold_942	19	Glutamyl-tRNA(Gln) amidotransferase subunit A, putative
SA_26172	scaffold_942	42	Glutamyl-tRNA(Gln) amidotransferase subunit A, putative
SA_26182	scaffold_942	3	Pyruvate kinase
SA_26184	scaffold_942	8	Threonine dehydratase/deaminase, putative
SA_26207	scaffold_942	10	Phenylalanine ammonia-lyase
SA_26219	scaffold_942	4	Protein binding protein, putative
SA_26292	scaffold_942	5	Dual specificity protein phosphatase, putative
SA_26340	scaffold_942	2	1-phosphatidylinositol-4,5-bisphosphate phosphodiesterase, putative
SA_26348	scaffold_942	5	Microsomal signal peptidase 25 kD subunit, putative
SA_26349	scaffold_942	4	Putative uncharacterized protein
SA_26361	scaffold_942	8	YIPF1-like protein
SA_26375	scaffold_942	2	Uncharacterized protein
SA_26381	scaffold_942	6	Putative uncharacterized protein
SA_26457	scaffold_942	3	AT5g04480/T32M21_80
SA_26503	scaffold_942	21	Katanin P80 subunit, putative
SA_26503	scaffold_942	3	Katanin P80 subunit, putative
SA_26517	scaffold_942	7	RNA binding protein, putative
SA_26559	scaffold_942	13	Uncharacterized protein
SA_26574	scaffold_942	1	Putative uncharacterized protein
SA_26574	scaffold_942	3	Putative uncharacterized protein
SA_26588	scaffold_942	15	Coiled-coil domain-containing protein MTMR15
SA_26625	scaffold_942	16	Synaptotagmin, putative
SA_26625	scaffold_942	17	Synaptotagmin, putative

SA_26642	scaffold_942	7	Uncharacterized protein
SA_26644	scaffold_942	1	Uncharacterized protein
SA_26644	scaffold_942	2	Uncharacterized protein
SA_26674	scaffold_942	3	Protein FAM119A
SA_26681	scaffold_942	3	Predicted protein
SA_26717	scaffold_942	36	Catalytic, putative
SA_26722	scaffold_942	7	Phytochrome-interacting factor, putative
SA_26757	scaffold_942	1	Protein COBRA, putative
SA_26832	scaffold_942	3	DNA binding protein, putative
SA_26847	scaffold_942	8	MscS family protein, putative
SA_26863	scaffold_942	1	Bromodomain protein
SA_26900	scaffold_942	4	Putative uncharacterized protein
SA_26905	scaffold_942	5	B2 protein, putative
SA_26945	scaffold_944	5	Receptor serine-threonine protein kinase, putative
SA_26945	scaffold_944	6	Receptor serine-threonine protein kinase, putative
SA_26945	scaffold_944	7	Receptor serine-threonine protein kinase, putative
SA_26951	scaffold_944	4	Auxin response factor-like protein
SA_26955	scaffold_944	16	Zinc finger protein, putative
SA_26955	scaffold_944	6	Zinc finger protein, putative
SA_26976	scaffold_944	1	YTH domain family protein
SA_27016	scaffold_944	1	Putative uncharacterized protein
SA_27081	scaffold_946	3	ARF GTPase activator, putative
SA_27156	scaffold_946	2	Putative uncharacterized protein
SA_27165	scaffold_946	15	ATP synthase gamma chain
SA_27188	scaffold_946	2	Pentatricopeptide repeat-containing protein, putative
SA_27200	scaffold_946	3	Homeobox protein, putative
SA_27230	scaffold_946	9	Kinase, putative
SA_27239	scaffold_946	3	SOUL-like protein (Fragment)
SA_27240	scaffold_946	4	Putative uncharacterized protein
SA_27288	scaffold_946	7	Predicted protein
SA_27316	scaffold_946	1	Predicted protein
SA_27349	scaffold_946	3	Nbs-lrr resistance protein
SA_27386	scaffold_946	4	Uncharacterized protein
SA_27390	scaffold_946	20	Fimbrin, putative
SA_27402	scaffold_948	8	Putative uncharacterized protein
SA_27447	scaffold_948	1	Putative uncharacterized protein
SA_27451	scaffold_948	3	Uncharacterized protein
SA_27451	scaffold_948	4	Uncharacterized protein
SA_27476	scaffold_948	17	Polycomb protein embryonic flower, putative
SA_27494	scaffold_948	6	Uncharacterized protein
SA_27510	scaffold_948	3	Amino acid binding protein, putative
SA_27514	scaffold_948	2	Ycf49-like protein
SA_27594	scaffold_948	1	Putative uncharacterized protein

SA_27622	scaffold_948	1	
SA_27627	scaffold_948	8	Silencing group A protein
SA_27689	scaffold_948	5	Methylenetetrahydrofolate reductase
SA_27694	scaffold_948	12	BRASSINOSTEROID INSENSITIVE 1-associated receptor kinase 1
SA_27705	scaffold_948	24	DRP
SA_27733	scaffold_948	4	Transcription factor bZIP10
SA_27735	scaffold_952	2	AT2G31560 protein
SA_27735	scaffold_952	4	AT2G31560 protein
SA_27758	scaffold_952	10	Putative uncharacterized protein
SA_27785	scaffold_954	9	Flavodoxin family protein
SA_27809	scaffold_954	2	Predicted protein
SA_27855	scaffold_954	24	Putative uncharacterized protein
SA_27868	scaffold_954	7	Putative uncharacterized protein
SA_27887	scaffold_954	5	Predicted protein
SA_27896	scaffold_954	5	2,4-dienoyl-CoA reductase, putative
SA_27929	scaffold_954	3	U2 snrnp auxiliary factor, small subunit, putative
SA_27929	scaffold_954	4	U2 snrnp auxiliary factor, small subunit, putative
SA_27933	scaffold_954	4	Hydroxysteroid dehydrogenase, putative
SA_27945	scaffold_954	5	Predicted protein
SA_27955	scaffold_954	4	Transmembrane emp24 domain-containing protein
SA_27957	scaffold_954	24	Ribosomal RNA small subunit methyltransferase B, putative
SA_27957	scaffold_954	30	Ribosomal RNA small subunit methyltransferase B, putative
SA_27957	scaffold_954	31	Ribosomal RNA small subunit methyltransferase B, putative
SA_27994	scaffold_954	2	DNA binding protein, putative
SA_28025	scaffold_954	1	3-hydroxyacyl-CoA dehyrogenase, putative
SA_28074	scaffold_954	1	DAG protein, chloroplast, putative
SA_28082	scaffold_954	2	Nbs-lrr resistance protein
SA_28134	scaffold_954	4	O-methyltransferase, putative
SA_28182	scaffold_954	5	Double-stranded DNA-binding protein
SA_28194	scaffold_954	8	Putative uncharacterized protein
SA_28216	scaffold_954	4	Putative uncharacterized protein
SA_28243	scaffold_954	11	Nitric-oxide synthase, putative
SA_28288	scaffold_954	7	Putative uncharacterized protein
SA_28335	scaffold_954	3	E3 ubiquitin ligase
SA_28359	scaffold_954	1	Predicted protein
SA_28391	scaffold_954	4	Pto kinase interactor
SA_28391	scaffold_954	9	Pto kinase interactor
SA_28438	scaffold_957	1	60S ribosomal protein L39 (Fragment)
SA_28485	scaffold_968	1	Glycosyltransferase QUASIMODO1, putative
SA_28538	scaffold_968	2	Dead box ATP-dependent RNA helicase, putative
SA_28561	scaffold_968	5	Uncharacterized protein

SA_28678	scaffold_983	17	V-H(+)-ATPase subunit A
SA_28719	scaffold_983	3	Zinc ion binding protein, putative
SA_28857	scaffold_983	3	Arginine/serine-rich splicing factor, putative
SA_28948	scaffold_983	8	Zinc finger A20 and AN1 domain-containing stress-associated protein
SA_28969	scaffold_995	6	Protein translocase, putative
SA_28971	scaffold_995	2	Putative uncharacterized protein
SA_28978	scaffold_995	3	Phi class glutathione transferase GSTF4
SA_29008	scaffold_995	1	Myosin heavy chain, clone, putative
SA_29028	scaffold_995	6	Clathrin assembly protein, putative
SA_29037	scaffold_995	10	Putative uncharacterized protein
SA_29037	scaffold_995	11	Putative uncharacterized protein
SA_29043	scaffold_995	11	Abc transporter, putative
SA_29091	scaffold_995	17	Sumo ligase, putative
SA_29161	scaffold_995	1	Zinc finger protein, putative
SA_29161	scaffold_995	2	Zinc finger protein, putative
SA_29189	scaffold_995	3	Gamma-tubulin complex component, putative
Alternative Donor			
SA_01740	scaffold_1102	5(0)-6(-25)	LOV/LOV protein
SA_02301	scaffold_1102	3(0)-4(+4)	Tobamovirus multiplication 2B
SA_05085	scaffold_1463	1(+429)-2(0)	Cysteine-type peptidase, putative
SA_06860	scaffold_24	2(0)-3(+23)	Putative uncharacterized protein
SA_07568	scaffold_267	5(+6)-6(0)	Uncharacterized protein
SA_07878	scaffold_270	7(0)-8(+138)	Uncharacterized protein
SA_08799	scaffold_330	5(+100)-6(0)	Predicted protein
SA_09312	scaffold_333	1(0)-2(+4)	AP2 domain class transcription factor
SA_09312	scaffold_333	1(0)-2(-66)	AP2 domain class transcription factor
SA_10463	scaffold_370	1(0)-2(-49)	Stress associated endoplasmic reticulum protein, putative
SA_10795	scaffold_379	1(+4)-2(0)	
SA_13434	scaffold_705	4(0)-5(+5)	Putative uncharacterized protein
SA_13500	scaffold_705	10(0)-11(+6)	Predicted protein
SA_14303	scaffold_795	5(-51)-6(0)	Poly-A binding protein, putative
SA_14838	scaffold_891	2(0)-3(+322)	Ubiquitin-conjugating enzyme E2
SA_17220	scaffold_913	1(-24)-2(0)	Uncharacterized protein
SA_18143	scaffold_916	5(0)-6(-24)	Predicted protein
SA_18143	scaffold_916	6(0)-7(+6)	Predicted protein
SA_18165	scaffold_916	4(+43)-5(0)	Serine/threonine protein kinase, putative
SA_18587	scaffold_917	1(-38)-2(0)	Transcriptional corepressor SEUSS, putative
SA_19840	scaffold_918	3(-12)-4(0)	Auxilin, putative
SA_21241	scaffold_922	1(0)-2(+31)	Thioredoxin I, putative
SA_21590	scaffold_923	2(0)-3(+14)	Acyl-peptide hydrolase-like protein
SA_22997	scaffold_927	1(-12)-2(0)	60S ribosomal protein L32, putative
SA_23285	scaffold_927	6(0)-7(+9)	Uncharacterized protein

SA_24239	scaffold_929	8(0)-9(+42)	Predicted protein
SA_26340	scaffold_942	2(0)-3(-104)	1-phosphatidylinositol-4,5-bisphosphate phosphodiesterase, putative
SA_28117	scaffold_954	1(+4)-2(0)	F3H9.10 protein
SA_28179	scaffold_954	9(+108)-10(0)	AML1
SA_28398	scaffold_954	2(0)-3(+32)	Protein binding protein, putative
Alternative acceptor			
SA_00264	scaffold_1017	10(0)-11(+9)	JMS09K11.7 protein
SA_00954	scaffold_1050	2(+3)-3(0)	Mak, putative
SA_01032	scaffold_1096	1(+275)-2(0)	mRNA, clone: RTFL01-47-M12
SA_02614	scaffold_1132	7(0)-8(+3)	Putative uncharacterized protein
SA_03871	scaffold_1290	12(0)-13(+3)	CIP73 protein
SA_04666	scaffold_1463	1(+6)-2(0)	Uncharacterized protein
SA_04732	scaffold_1463	2(0)-3(+3)	Putative uncharacterized protein
SA_05489	scaffold_218	2(+14)-3(0)	Zinc finger protein, putative
SA_05832	scaffold_219	22(-15)-23(0)	Uncharacterized protein
SA_05912	scaffold_219	3(+3)-4(0)	Uncharacterized protein
SA_06603	scaffold_24	8(0)-9(+5)	Predicted protein
SA_07010	scaffold_244	8(-4)-9(0)	At1g01050/T25K16_5
SA_07037	scaffold_244	4(-31)-5(0)	Ubiquitin-conjugating enzyme E2
SA_07582	scaffold_267	9(-31)-10(0)	F-box family protein
SA_07958	scaffold_270	5(0)-6(-6)	Methylosome subunit pICln, putative
SA_08648	scaffold_301	11(+24)-12(0)	Bile acid Na+ symporter family protein
SA_08740	scaffold_330	1(-85)-2(0)	Snare protein ykt6, putative
SA_09948	scaffold_350	6(+3)-7(0)	Putative uncharacterized protein
SA_10447	scaffold_370	3(0)-4(+112)	Cdk10/11, putative
SA_11097	scaffold_394	15(0)-16(+513)	T-complex protein 1 subunit alpha
SA_12020	scaffold_400	3(0)-4(-15)	Putative uncharacterized protein
SA_12622	scaffold_407	10(+13)-11(0)	RNA-binding protein, putative
SA_14324	scaffold_795	31(+3)-32(0)	Ubiquitin-protein ligase, putative
SA_15015	scaffold_893	5(0)-6(+8)	Homeobox protein knotted-1-like 3
SA_15905	scaffold_897	2(-10)-3(0)	Glycosyltransferase QUASIMODO1, putative
SA_16411	scaffold_899	3(0)-4(+3)	Type 2 histone deacetylase a
SA_16454	scaffold_899	9(-5)-10(0)	Ascorbate peroxidase
SA_16791	scaffold_899	4(+3)-5(0)	Nuclear acid binding protein, putative
SA_17717	scaffold_916	1(0)-2(+3)	Putative uncharacterized protein
SA_19013	scaffold_917	2(0)-3(+12)	Putative uncharacterized protein
SA_19063	scaffold_918	1(-9)-2(0)	Non-imprinted in Prader-Willi/Angelman syndrome region protein, putative
SA_19249	scaffold_918	6(0)-7(-6)	Tfiif-alpha, putative
SA_19577	scaffold_918	1(-3)-2(0)	Uncharacterized protein
SA_21389	scaffold_923	4(0)-5(-3)	Putative uncharacterized protein
SA_21494	scaffold_923	7(0)-8(+3)	Calmodulin binding protein, putative
SA_21549	scaffold_923	5(0)-6(+3)	Predicted protein (Fragment)

SA_21901	scaffold_923	2(0)-3(-15)	DNA binding protein, putative
SA_22415	scaffold_925	2(0)-3(+3)	Eukaryotic peptide chain release factor GTP-binding subunit, putative
SA_22710	scaffold_925	15(+13)-16(0)	CBL-interacting protein kinase 12
SA_22876	scaffold_927	7(0)-8(+3)	Uncharacterized protein
SA_23293	scaffold_927	4(0)-5(+3)	Putative uncharacterized protein
SA_24543	scaffold_930	3(0)-4(+3)	RING finger protein, putative
SA_24734	scaffold_930	15(+3)-16(0)	ATP binding protein, putative
SA_25163	scaffold_930	2(0)-3(+202)	Light-inducible protein atls1, putative
SA_25593	scaffold_934	6(0)-7(-3)	Lysine--tRNA ligase
SA_25663	scaffold_934	5(0)-6(+6)	Predicted protein
SA_25849	scaffold_934	1(+3)-2(0)	Predicted protein
SA_26631	scaffold_942	10(+79)-11(0)	Eukaryotic peptide chain release factor GTP-binding subunit, putative
SA_26768	scaffold_942	1(-3)-2(0)	Putative uncharacterized protein
SA_26976	scaffold_944	8(-15)-9(0)	YTH domain family protein
SA_27270	scaffold_946	3(+3)-4(0)	Chromatin remodeling complex subunit
SA_27805	scaffold_954	5(0)-6(-18)	JHL18I08.4 protein
SA_28283	scaffold_954	3(+3)-4(0)	Tripeptidyl peptidase II, putative
SA_28303	scaffold_954	9(0)-10(+6)	Chaperone binding protein, putative
SA_28598	scaffold_983	3(0)-4(+4)	Putative uncharacterized protein
SA_28952	scaffold_983	1(0)-2(+3)	60S acidic ribosomal protein
SA_28952	scaffold_983	2(0)-3(-48)	60S acidic ribosomal protein
Alternative position			
SA_00364	scaffold_1030	1(+2)-2(+539)	Beta-ureidopropionase, putative
SA_02663	scaffold_1132	7(+2)-8(-1)	EBP1
SA_11233	scaffold_394	2(+431)-3(+73)	Nucleotide binding protein, putative
SA_12137	scaffold_400	8(+4)-9(-4)	Proteasome subunit alpha type
SA_17202	scaffold_913	1(+405)-2(+236)	Endosomal P24A protein, putative
SA_25677	scaffold_934	6(-1)-7(+2)	Cellulose synthase A catalytic subunit 6 [UDP-forming], putative
SA_26976	scaffold_944	8(+2)-9(-1)	YTH domain family protein
SA_28726	scaffold_983	12(+118)-13(+186)	mRNA, clone: RTFL01-22-B23

The legend "Exon pair" is for exon skipping; "No. of retained intron" is for intron retention; "Splice" is for alternative donor, acceptor and position.

No. is short for number.

Supplementary Table S4. Distributions of alternative splicing (AS) types for evolutionarily conserved AS (ECAS) events among four *Sonneratia* species and non-ECAS events.

	ES	IR	AltD	AltA	AltP	Total	Number of genes
ECAS							
Frequencies	99	1161	30	57	8	1355	1170
Proportions (%)	7.31	85.68	2.21	4.21	0.59		
non-ECAS							
Frequencies	286	3465	707	1064	371	5893	4117
Proportions (%)	4.85	58.8	12	18.06	6.3		
G-test (on ECAS vs non-ECAS)							
P-value	$4.8*10^{-4}$ ***	$< 2.2*10^{-16}$ ***	$< 2.2*10^{-16}$ ***	$< 2.2*10^{-16}$ ***	$< 2.2*10^{-16}$ ***		

ES, exon skipping; IR, intron retention; AltD, alternative donor; AltA, alternative acceptor; AltP, alternative position.

***, $P < 0.001$.

Supplementary Table S5. Distribution of alternative splicing (AS) positions in evolutionarily conserved AS (ECAS) events and non-ECAS events.

	Coding region*	5' UTR	3' UTR	Total
ECAS				
Frequencies	202	11	23	236
Proportions (%)	85.59	4.66	9.75	
non-ECAS				
Frequencies	2244	206	218	2268
Proportions (%)	84.11	7.72	8.17	
G-test (on ECAS vs non-ECAS)				
P-value	0.544	0.068	0.411	

No., number.

* The AS position is assigned as "in coding region" if the AS is in the coding region of constitutive isoform.

Only genes containing only one AS events were taken into account.

Supplementary Table S6. Domains affected by evolutionarily conserved AS (ECAS) events.

Gene ID	Domain predicted	Constitutive isoform			Alternative isoform			Modification prediction
		No. of domain	Star position	Domain length	No. of domain	Start position	Domain length	
Exon skipping								
SA_01999	Auxin_BP	1	71	167	1	55	168	Modified
SA_02727	TPR_11	1	415	62	0	-	-	Lost
SA_03574	Pollen_Ole_e_I	1	136	106	1	136	79	Modified
SA_04145	PDH_E1_alph_y	1	64	316	1	64	165	Modified
SA_29198	EF-hand_8	1	58	50	0	-	-	Lost
	EF-hand_7	1	118	61	0	-	-	Lost
Intron retention								
SA_00412	DUF1682	1	68	138	0	-	-	Lost
SA_00627	ArfGap	1	456	108	0	-	-	Lost
SA_01670	FAD_binding_8	1	534	75	0	-	-	Lost
SA_01714	K_trans	1	102	541	1	102	574	Modified
SA_01894	EamA	2	537, 738	114, 73	0	-	-	Lost
SA_01949	Di19_C	1	60	91	0	-	-	Lost
SA_03279	V-SNARE_C	1	151	65	0	-	-	Lost
SA_03546	K_trans	1	26	580	1	26	401	Modified
SA_03841	Ion_trans	1	109	244	1	109	271	Modified
SA_04747	Pkinase_Tyr	0	-	-	1	539	263	Gained
SA_04759	Ribosomal_L17	1	77	97	0	-	-	Lost
SA_05138	HSP90	1	184	163	1	184	516	Modified
SA_05172	HSP70	1	72	315	1	72	483	Modified
SA_05304	dsrm	2	97, 183	66, 63	0	-	-	Lost

SA_05702	DUF1218	1	651	98	0	-	-	Lost
SA_06623	AP2	1	331	51	0	-	-	Lost
SA_07221	KNOX1	1	834	43	0	-	-	Lost
	KNOX2	1	895	55	0	-	-	Lost
	Homeobox_KN	1	1043	40	0	-	-	Lost
SA_07415	CPSase_L_D2	1	365	72	0	-	-	Lost
SA_07708	Lipase_GDSL	1	1326	322	0	-	-	Lost
SA_09436	Ras	1	19	159	0	-	-	Lost
SA_09509	PPR_2	5	569, 673, 848, 920, 1094	49, 50, 50, 46, 49	0	-	-	Lost
SA_09964	Aldo_ket_red	1	45	54	0	-	-	Lost
SA_09998	Lipase_GDSL	1	35	313	0	-	-	Lost
SA_10231	HSP70	1	18	347	1	18	486	Modified
SA_10537	Pkinase	0	-	-	1	363	267	Gained
SA_11140	AAA_11	1	173	212	0	-	-	Lost
	AAA_12	1	393	197	0	-	-	Lost
SA_11439	CTP_transf_1	1	110	254	1	117	285	Modified
SA_11657	Methyltransf_4	1	85	108	0	-	-	Lost
SA_11707	DnaJ	1	260	64	0	-	-	Lost
	CTDII	1	515	79	0	-	-	Lost
SA_12154	ZZ	1	647	42	0	-	-	Lost
	Myb_DNA-binding	1	707	42	0	-	-	Lost
	SWIRM	1	1073	67	0	-	-	Lost
SA_12591	Ribosomal_S9	1	253	101	1	253	124	Modified
SA_12688	Prp19_bind	1	337	280	0	-	-	Lost
SA_12712	DUF3774	1	257	28	0	-	-	Lost
SA_13210	RRM_5	1	311	57	0	-	-	Lost

SA_13259	zf-RING_2	1	885	44	0	-	-	Lost
SA_13261	PP2C	1	422	251	0	-	-	Lost
SA_13404	PIG-H	1	687	64	0	-	-	Lost
SA_13996	Kelch_1	0	-	-	1	195	46	Gained
SA_15438	MRP-L46	1	139	51	0	-	-	Lost
SA_15826	DRMBL	0	-	-	1	220	128	Gained
SA_16049	Methyltransf_29	1	372	501	0	-	-	Lost
SA_16604	RRM_1	1	148	58	0	-	-	Lost
SA_16732	DUF868	1	81	279	0	-	-	Lost
SA_18615	TCP	1	909	83	0	-	-	Lost
SA_20692	TRAM_LAG1_CLN8	1	628	196	0	-	-	Lost
SA_20959	Fasciclin	1	156	112	0	-	-	Lost
SA_21404	SufE	1	91	91	0	-	-	Lost
	BolA	1	279	67	0	-	-	Lost
SA_21458	Ribophorin_I	2	52, 182	111, 92	1	52	111	Lost
SA_22389	MutS_III	1	299	246	1	299	104	Modified
SA_22988	ATP_bind_1	1	30	244	0	-	-	Lost
SA_24205	Pkinase_Tyr	1	1095	84	0	-	-	Lost
	S_locus_glycop	1	787	112	0	-	-	Lost
	PAN_2	1	935	61	0	-	-	Lost
	B_lectin	1	652	110	0	-	-	Lost
SA_25130	THF_DHG_CYH	1	887	117	0	-	-	Lost
	THF_DHG_CYH_C	1	1006	114	0	-	-	Lost
SA_25141	DUF3411	1	103	169	0	-	-	Lost
SA_25964	DUF239	1	867	181	0	-	-	Lost
SA_25973	CCT	1	822	38	0	-	-	Lost

SA_27165	ATP-synt	1	907	146	0	-	-	Lost
SA_27705	DLP_1	1	750	263	0	-	-	Lost
	PH_RhoGap25-like	1	1280	124	0	-	-	Lost
	GED	1	1449	74	0	-	-	Lost
SA_27809	TFIIF_beta	1	43	47	0	-	-	Lost
SA_27955	EMP24_GP25L	1	472	173	0	-	-	Lost
SA_28182	dsDNA_bind	1	9	111	1	9	85	Modified
SA_28194	Cullin	1	469	199	0	-	-	Lost
	Cullin_Nedd8	1	695	64	0	-	-	Lost
SA_28335	Sina	1	240	200	0	-	-	Lost
Alternative acceptor								
SA_19249	TFIIF_alpha	1	48	400	1	48	398	Modified
SA_27805	CLTH	1	77	149	1	77	143	Modified

Supplementary Table S7. Gene ontology (GO) enrichment analysis of evolutionarily conserved AS (ECAS) genes in *Sonneratia*.

GO ID	Term Annotation	No. of conserved AS genes	*P*-value
Cell Component			
GO: 0009706	chloroplast inner membrane	4	0.012
GO: 0008180	signalosome	3	0.013
GO: 0033178	proton-transporting two-sector ATPase cocomplex, catalytic domain	5	0.021
GO: 0005802	trans-Golgi network	6	0.025
GO: 0005794	Golgi apparatus	27	0.029
GO: 0000145	Exocyst	3	0.043
Molecular Function			
GO: 0043565	sequence-specific DNA binding	12	0.001
GO: 0046961	proton-transporting ATPase activity, rotational mechanism	5	0.004
GO: 0003712	transcription cofactor activity	5	0.011
GO: 0035091	phosphatidylinositol binding	5	0.011
GO: 0008270	zinc ion binding	41	0.021
GO: 0005097	Rab GTPase activator activity	5	0.024
GO: 0004222	metalloendopeptidase activity	5	0.043
GO: 0016905	myosin heavy chain kinase activity	3	0.046
GO: 0005509	calcium ion binding	16	0.046
Biological Process			
GO: 0032940	secretion by cell	8	0.003
GO: 0046474	glycerophospholipid biosynthesis process	8	0.003
GO: 0006119	oxidative phosphorylation	11	0.008
GO: 0006777	Mo-molybdopterin cofactor biosynthesis process	3	0.013
GO: 0045488	pectin metabolic process	3	0.013
GO: 0006662	glycerol ether metabolic process	5	0.02
GO: 0032851	positive regulation of Rab GTPase activity	5	0.02
GO: 0045935	positive regulation of nucleobase-containing compound metabolic process	6	0.036
GO: 0015991	ATP hydrolysis coupled proton transport	6	0.039
GO: 0010498	proteasomal protein catabolic process	3	0.042
GO: 0045036	protein targeting to chloroplast	3	0.042

No. is short for number.

Supplementary Table S8. Functional annotation of the evolutionarily conserved AS (ECAS) genes assigned to Gene ontology (GO) terms ATP hydrolysis coupled proton transport (GO: 0015991), calcium ion binding (GO: 0005509) and oxidative phosphorylation (GO: 0006119) in *S. alba*. Genes assigned to more than one GO terms were list only once.

Gene ID	Accession number of Arabidopsis	Gene description
proton-transporting ATPase activity, rotational mechanism (GO: 0046961)		
SA_07404	At2g07698	ATPase, F1 complex, alpha subunit protein
SA_12122	At3g42050	vacuolar ATP synthase subunit H family protein
SA_14841	At4g11150	*VHA-E1*, vacuolar H^+-ATPase subunit E isoform 1
SA_18019	At4g02620	vacuolar ATPase subunit F family protein
SA_28678	At1g78900	*VHA-A*, vacuolar ATP synthase subunit A
ATP hydrolysis coupled proton transport (GO: 0015991)		
SA_12345	At2g21410	*VHA-A2*, vacuolar proton ATPase subunit VHA-a isoform 2
calcium ion binding (GO: 0005509)		
SA_03107	At4g20260	*PCAP1*, plasma-membrane associated cation-binding protein
SA_04445	At4g25000	*AMY1*, alpha-amylase-like
SA_04886	At1g27520	*MNS5*, mannosidase 5
SA_05088	At3g20410	*CDPK9*, calcium dependent protein kinase 9
SA_06082	At3g55940	*PLC15*, phosphoinositide-specific phospholipase C 15
SA_12609	At5g24270	*SOS3*, salt overly sensitive 3/calcineurin B-like protein 4
SA_13527	At3g50530	*CRK*, CDPK-related kinase
SA_13832	At5g61790	*CNX1*, calnexin 1
SA_14971	At3g50530	*CRK*, CDPK-related kinase
SA_18783	At3g50530	*CRK*, CDPK-related kinase
SA_19886	At1g51590	*MNS1*, alpha-mannosidase I
SA_20225	At4g01710	*ARPC5*, actin related protein 2/3 complex, subunit 5A
SA_21449	At1g76450	Photosystem II reaction center PsbP family protein
SA_26340	At3g08510	*PLC2*, phosphoinositide-specific phospholipase C 2
SA_27390	At4g26700	*FIM1*, fimbrin-like protein
SA_29198	At5g21274	*CAM6*, calmodulin 6
oxidative phosphorylation (GO: 0006119)		
SA_05616	At2g18230	*PPA2*, pyrophosphorylase 2
SA_07010	At1g01050	*PPA1*, pyrophosphorylase 1
SA_07445	At2g18960	*PMA*, plasma membrane proton ATPase
SA_10493	At5g13440	ubiquinol-cytochrome C reductase iron-sulfur subunit
SA_26130	At1g78920	*VHP2*, vacuolar H^+-pyrophosphatase 2

Supplementary Table S9. Functional annotation of the evolutionary conserved AS (ECAS) genes in response to osmotic, oxidative and heat stress in *S. alba*. Genes assigned to more than one GO terms were list only once.

Gene ID	Accession number of Arabidopsis	Gene description
response to osmotic stress (GO: 0006970)		
SA_03951	At5g10860	*CBSX3*, cystathionine beta-synthase (CBS) domain-containing protein
SA_08955	At5g43330	cytosolic-NAD-dependent malate dehydrogenase 2
SA_12800	At2g32060	40S ribosomal protein S12
SA_12966	At2g23350	*PAB4*, poly(A) binding protein 4
SA_14841	At4g11150	*VHA-E1*, vacuolar H^{+}-ATPase subunit E isoform 1
SA_21670	At4g24190	*HSP90.7*, heat shock protein 90.7
SA_22710	At1g01140	*CIPK9*, CBL-interacting protein kinase 9
SA_24912	At5g19330	*ARIA*, arm repeat protein interacting with ABF2
SA_25246	At3g57290	*EIF3E*, eukaryotic translation initiation factor 3E
SA_00745	At1g05260	*RCI3*, Rare Cold Inducible gene 3
SA_25677	At4g32410	*CESA1*, cellulose synthase 1
SA_12609	At5g24270	*SOS3*, salt overly sensitive 3/calcineurin B-like protein 4
SA_01447	At2g43020	*PAO2*, polyamine oxidase 2
SA_10597	At4g37220	cold acclimation protein WCOR413 family
SA_18770	At3g50830	cold acclimation protein WCOR413-like protein beta form
response to oxidative stress (GO: 0006979)		
SA_20388	At3g16950	*LPD1*, lipoamide dehydrogenase 1
SA_07119	At2g35380	peroxidase superfamily protein
SA_07415	At1g65820	microsomal glutathione s-transferase
SA_14968	At1g49670	*NQR*
SA_16454	At1g07890	*APX1*, ascorbate peroxidase 1
SA_21357	At5g55070	dihydrolipoamide succinyltransferase
SA_07693	-	alpha-crystallin domain of heat shock protein-containing protein
SA_16621	At1g11890	*SEC22*, secretion 22
SA_18560	At1g22770	*GI*, gigantea
response to heat (GO: 0009408)		
SA_07247	At4g23100	*GCL*, glutamate-cysteine ligase
SA_10773	At1g77000	*SKP2B*, Arabidopsis homolog of homolog of human SKP2 2

Supplementary Table S10. Gene IDs, AS types, primer sequences of the genes used for Reverse transcription-PCR (RT-PCR) validation.

Gene ID	AS types	Length of constitutive isoform	Length of AS isoform	Primer sequence (From5' to 3')
SA_29008	IR	550	640	F: CCCCCAAGCTCCCACACTC<br>R: GGGAAGCACAGGCGGAGAT
SA_05564	IR	287	397	F: GCGGTGTCCTGACGGCGTTTAC<br>R: TAGGCTGATCCGTGGTTGGTTCT
SA_06314	IR	358	497	F: AGCCGTTAATTCAAAGACCACTG<br>R: CGCCAGGATGAAAAGGAGCTC
SA_28673	ES	291	165	F: CGGGAACATGATCAAGCGAGAG<br>R: GGGCCAGCTGCAGTTTTTCTAG
SA_03574	ES	262	180	F: GCCGCTTTGGTTTCGAGAC<br>R: AACGGACGGTGCAAAGCTC
SA_29198	ES	337	294	F: GGTCGTCGGTGAGCTGGTCT<br>R: ACGGTACCATCGATTTCCCAGAG
SA_01949	IR	614	713	F: TTCGACTGAGTTGCTTGTTAGGTTCCT<br>R: TCCGTGAAGATCTATGGTATTGCTCCC
SA_09436	IR	201	286	F: GGCCCAGTTGTACGGTCGCTTTTTGA<br>R: GGGTTGGGCTGATGACGCTGGTGT
SA_17996	IR	186	292	F: ATGCGAACTTCCCCTCACCCAAAATCT<br>R: CCCCGCTGGTCAAAAGTCTCCTCCT
SA_27868	IR	275	365	F: ATGCAGAGGCCCCGCTTTTATC<br>R: TGGGCTGGGCTTTGCTTCACG
SA_25369	IR	278	443	F: GCCCGCCTGCACCAAAGAAACC<br>R: CCGGCATCTGTCATCAAGCTCATCTGT
